# Multidrug-resistant *E. Coli* in wastewater sources: a comparative study and identification of resistance hotspots

**DOI:** 10.1186/s12866-025-04244-5

**Published:** 2025-08-12

**Authors:** Reham R. Abdelgalel, Reham Ali Ibrahem, Doaa Safwat Mohamed, Abo Bakr F. Ahmed

**Affiliations:** 1 Department of Microbiology and Immunology, Faculty of Pharmacy, Minia University, Minia, Egypt; 2 Department of Microbiology and Immunology, Faculty of Pharmacy, Sohag University, Sohag AlGadIda City, Egypt

**Keywords:** Wastewater, Beta lactamases, Phylogenetic typing, Diarrheagenic *E. coli*

## Abstract

**Background:**

This work aims to study the patterns of resistance to antibiotics in wastewater samples collected from hospitals, the community, and the wastewater treatment plant using the indicator bacterium *Escherichia coli*. In addition, studying the phylogenetic type and screening for beta-lactamase-producing and diarrhoeagenic *E. coli*.

**Methods:**

The isolates were subjected to antibiotic sensitivity tests using the Kirby-Bauer disc diffusion method using 11 antibiotics. Conventional PCR was performed for the detection of *E. coli* that produces beta-lactamase, as well as for the phylogenetic typing of isolates and to detect diarrheagenic strains.

**Results:**

From 120 wastewater samples, 92 *E. coli* strains were recovered and among them 66.3% were resistant. The highest resistance of isolates was observed in ampicillin/sulbactam with a moderately high rate of 37%, while the lowest resistance was reported in gentamycin at a low rate of 3.3%. Hospital wastewater isolates (HW) (75%) were more resistant than community wastewater isolates (CW) (50%). A high rate of resistance was reported in tetracycline (46.9%) among the hospital wastewater isolates, while a moderate rate of resistance was detected among community wastewater isolates to aztreonam (22.2%), making them the least effective antibiotics in these groups. More resistant isolates were found in WWTP influent (83.3%) than in effluent (44.4%). However, effluent isolates showed higher resistance to certain antibiotics, including cefepime (11.1% vs. 8.3%), piperacillin/tazobactam (11.1% vs. 4.2%), and imipenem (5.6% vs. 4.2%), than the influent. *E. coli* strains that showed MDR phenotype were 42.6% of resistant strains, with higher prevalence among HW (50%) and WWTPs influent (45%) than WWTPs effluent (37.5%) and CW (22.2%). Although resistance to Access antibiotics was the highest across all sources, it was comparable to the resistance observed for Watch antibiotics. At least one of the tested beta-lactamase genes was detected in 67.39% of beta-lactam-resistant *E. coli* strains, with *bla*_*TEM*_ (62.1%) and *bla*_*OXA−48*_ (24.1%) being the highest prevalent among ESBLs and carbapenemase genes, respectively. About 83.69% of strains were commensal, while only 16.26% were pathogenic, in the form of enterotoxigenic *E. coli*; diarrheagenic *E. coli* was detected in 4.26% of strains.

**Conclusion:**

Even though resistance was less in community wastewater than hospital wastewater, community outlets might be a source of spreading resistance. Furthermore, wastewater treatment might not be enough to eliminate resistant bacteria from the effluent; as a result, the effluent quality should be continuously monitored and evaluated.

**Supplementary Information:**

The online version contains supplementary material available at 10.1186/s12866-025-04244-5.

## Background

In today’s medical practices, increased microbial resistance to commonly used antibiotics has become a serious concern. The primary compounds and their active metabolites, antibiotic-resistant bacteria, and their resistant genes are frequent pollutants in various environments due to years of antibiotic overuse. Wastewater, as well as human and animal feces, are known to be significant sources of resistant bacteria [1]. Moreover, wastewater is a crucial site for the horizontal transfer of resistant genes and the selection for antibiotic resistance [2–4]. Hospital wastewater (HW), in particular, contains a large number of resistant bacteria because several antibiotics are used in hospitals and are disposed of in wastewater [5]. Besides, antibiotic usage at home can lead to resistance in the microbiomes of the gut and other microbiomes due to abuse, incorrect use, or insufficient therapy [6]. Research on the significance of the normal microbiota of humans as a source of resistant bacteria is limited due to the difficulty of collecting large fecal samples from healthy individuals. So, measuring resistance to antibiotics in commensal indicators like *Escherichia coli* isolated from community wastewater (CW) specimens is an alternative method [7]. Additionally, this method can provide an early warning by detecting new or rare patterns of resistance in an area [7, 8]. Thus, it’s very significant to inspect other wastewater outlets, besides hospitals, that can contain antibiotic-resistant bacteria [9].

In addition, wastewater treatment plants act as reservoirs, receiving, harboring, and spreading resistant bacteria and their resistant genes. However, there is still not sufficient proof that WWTPs, particularly biological treatment techniques, contribute to the spread of antibiotic resistance. According to certain research, WWTPs can significantly reduce the amount of resistant bacteria in the effluent [10, 11]. However, further studies indicate that WWTPs are crucial sites for the spread of these resistant bacteria and genes [12]. Therefore, additional studies and evaluations must be performed to examine the involvement of WWTPs in the development or reduction of antibiotic resistance.

**Fig. 1:**
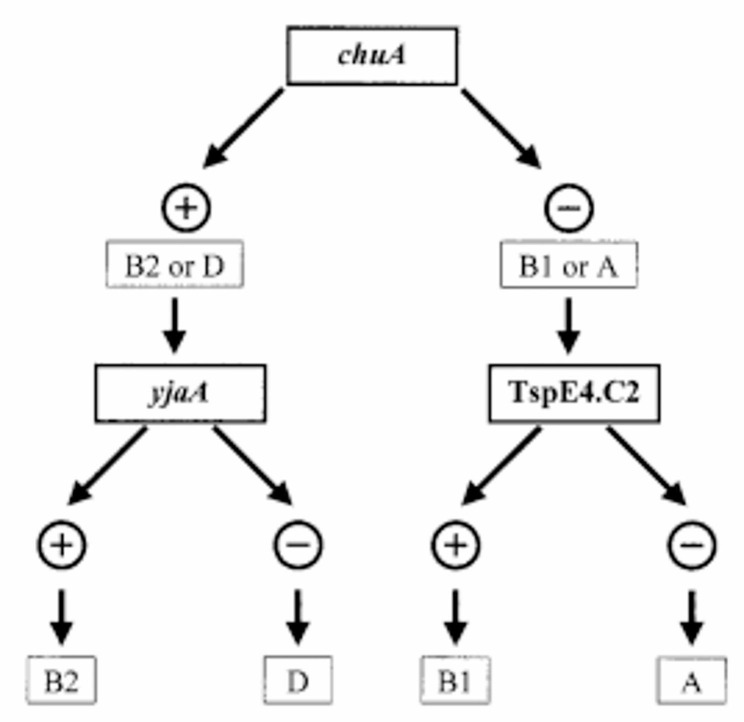
Dichotomous decision tree utilized for the determination of the phylogenetic group of *E. coli* strains [16]

*E.coli* is a bacterial species found in wastewater, and it is frequently utilized as an indicator for fecal pollution in the aquatic environment [13]. *E. coli* is prevalent naturally in the gastrointestinal system and can be detected in feces from diseased and convalescent patients, as well as in poorly managed wastewater [14]. Furthermore, several intestinal and extra-intestinal illnesses can be produced by *E.coli*, including diarrhea, septicemia, urinary tract infections, and neonatal meningitis [15]. Phylogenetic analysis can classify *E. coli* strains into the following major groups: A, B1, B2, and D. In this classification, strains of groups A and B1 are commensals, whereas groups B2 and D are pathogenic [16]. Certain genes or DNA segments may serve as markers for these phylogenetic analysis as shown in (Fig. 1) which includes (i) *chuA*, a gene in enterohemorrhagic O157:H7 *E. coli*, necessary for heme transport; (ii) *yjaA*, a gene with uncertain purpose that initially indicated in a more recent full genome sequence of *E. coli* K-12; and (iii) an unidentified DNA fragment called TspE4.C2 [16].

Moreover, multiple epidemiological studies have established a strong association between diarrhea and five pathotypes of *E.coli*, enteroinvasive (EIEC), Shiga toxin-producing (STEC), enteropathogenic (EPEC), enterotoxigenic (ETEC), and enteroaggregative (EAEC) [17]. These categories of *E. coli* are represented by *eae* and *bfpA* genes for (EPEC), *CVD432* probe for (EAEC), *ipaH* gene for (EIEC), *heat-stable toxin (ST)* and *heat-labile toxin (LT)* genes for (ETEC), *stx1* and *stx2* genes for (STEC) [18].

*E. coli* strains show resistance to a variety of antibiotic classes with various mechanisms of action [19] Including β-lactam antibiotics that are considered an essential therapy for many gram-positive and gram-negative pathogens [20]. More than 50 licensed medications are involved in this class, including penicillins, cephalosporins, carbapenems, and monobactams, and these agents account for more than 65% of the global antibiotic market [21]. Accordingly, the spread of resistance to such antibiotics is an important threat to public health.

The resistance of *E. coli* to several β-lactam antibiotics is mainly due to the production of β-lactamases [19]. Many resistance genes found on plasmid and chromosomal DNA express these β-lactamases [22]. *Bla*_*TEM*_, *bla*_*SHV*,_ and *bla*_*CTX−M*_ are genes encoding the most common extended-spectrum beta-lactamases among Enterobacteriaceae spp [23]. Carbapenems have become antimicrobial drugs of last resort in the treatment of ESBL-producing *E. coli*; nevertheless, resistance to carbapenems among Enterobacteriaceae has been developed worldwide as a result of the acquisition of genes like *bla*_*NDM*,_ *bla*_*KPC*_, *bla*_*VIM*_, *bla*_*IMP*_, and *bla*_*OXA−48*_ which are referred as carbapenemases [24]. Wastewater was revealed as a source of beta-lactamase-producing *E. coli* and other antibiotic-resistant bacteria (ARB) [25]. These resistant genes are strongly associated with transmissible genetic elements that can promote resistance to other bacteria by horizontal transfer of genes, which may be enhanced by numerous factors prevalent in wastewater [26].

Therefore, in this work, we aim to analyze the antimicrobial resistance patterns of *E. coli* in wastewater specimens obtained from hospitals, community sources, and WWTPs to enhance our understanding of the presence and dissemination of antimicrobial resistance in the living populations. Besides, comparing the antibiotic resistance levels among hospital and community outlets to determine if either of these two outlets acts as a source for the dispersion of resistant bacteria. Additionally, by comparing the resistance levels of WWTP influents and effluents, we can find out how the wastewater treatment process affects the spread of resistant bacteria.

## Methods

### Sample collection

A total of 120 wastewater samples were collected from various sources in Sohag City, Egypt which has a population of approximately 619,854 residents as follows: (1) Hospital wastewater samples (*n* = 49), collected from the outlets of three different hospitals (Sohag University Hospital, Masr Hospital, Tiba Royal Hospital); (2) Community wastewater samples (*n* = 28), obtained from the outlets of various residential homes, Sohag University, and the university student dormitory; (3) Al Kawlah wastewater treatment plant samples (*n* = 43), including 29 samples from the influent and 14 samples from the effluent after tertiary treatment. A map for different collection places is in the supplementary data. Each grab sample was collected in a sterile 100 mL plastic bottle (BoenMed, China), transported to the laboratory in a cold container with ice packs, and processed within a maximum of 6 h post-collection. Sampling was conducted from January 2021 to November 2023.

### Isolation and identification of *E. coli*

Each sample was thoroughly mixed, and then cultivated on the following selective media: MacConkey agar (TM Media, India; Lot No. MG329CT01), Eosin Methylene Blue (EMB) agar (HiMedia, India; Lot No. 0000391538), Cetrimide agar (HiMedia, India; Lot No. 0000317967), Mannitol Salt agar (Oxoid, UK), and Salmonella-Shigella (S-S) agar (HiMedia, India), followed by incubation at 37 °C for 24 h. The appearance of pink colonies on MacConkey agar indicated the presence of lactose-fermenting gram-negative bacteria. Colonies showing a green metallic sheen on EMB agar were selected for further biochemical characterization, including tests for oxidase activity, fermentation of lactose, sucrose, and glucose, indole production, citrate utilization, urea hydrolysis, motility, hydrogen sulfide production, and tryptophan deamination.

### Testing of antibiotic sensitivity

The standard Kirby Bauer disc diffusion method was used for antibiotic sensitivity testing of *E.coli* strains using 11 antibiotics (HiMedia, India) from 8 classes that were ampicillin/sulbactam (10/10 µg) (β -lactam combination), tetracycline (30 µg) (tetracycline), piperacillin/tazobactam (100/10 µg) (B-lactam combination), gentamycin (10 µg) (aminoglycoside), ceftriaxone (30 µg) (cephem), levofloxacin (5 µg) (quinolone), trimethoprim/sulphamethoxazole (1.25/23.75 µg) (folate pathway antagonist), cefepime (30 µg) (cephem), chloramphenicol (30 µg) (phenicol), imipenem (10 µg) (carbapenem) and aztreonam (30 µg) (monobactam).

Between three to five colonies from overnight cultures grown on nutrient agar were aseptically collected and suspended in 3–4 mL of sterile saline. The turbidity of the resulting bacterial suspension was adjusted to match the 0.5 McFarland standard (HiMedia, India). Antimicrobial susceptibility was determined by measuring the inhibition zone diameters, which were then interpreted as sensitive, intermediate, or resistant based on the Clinical and Laboratory Standards Institute (CLSI) guidelines (2020).

### Extraction of DNA

For DNA extraction from *E. coli* strains, the boiling method was used, where two overnight-growth colonies of isolates were put in a test tube with 1 ml of distilled water, then boiled in a water bath for 10 min. After that, centrifugation was done for 5 min at 1000 rpm. Five microliters of supernatant were utilized for the PCR [27]. The concentration and purity of the extracted DNA were evaluated using the NanoDrop spectrophotometer.

### Reaction mixture for PCR

PCR assays were carried out in a Veriti thermal cycler (Thermo Fisher Scientific, US) using a 25-µL reaction mixture, containing, in the case of multiplex reaction, 3 µL of DNA template, 10 µL of PCR Master Mix (Solis Biodyne, Tartu, Estonia), 2 µL of distilled water, and 1 µL each of 20 pmol forward and reverse primers. While triplex PCR was done using 3 µL of DNA template, 12 µL of PCR Master Mix, 4 µL of distilled water, and 1 µL each of 20 pmol forward and reverse primers. Duplex PCR was done for the detection of *bla*_*IMP*_ and *bla*_*VIM*_ genes using 12 µL of PCR Master Mix, 3 µL of template DNA, 1 µL of each primer, and 6 µL of distilled water.

### Detection of beta-lactamase-producing *E. coli*

Conventional PCR was used for the detection of ESBLs (*bla*_*TEM*_, *bla*_*SHV*_, *bla*_*CTX−M*_) and carbapenemase genes (*bla*_*IMP*_, *bla*_*VIM*_, *bla*_*KPC*_, *bla*_*NDM*_, *bla*_*OXA−48*_) among *E. coli* strains that showed resistance to at least one of the used beta-lactam antibiotics (ampicillin/sulbactam, aztreonam, cefepime, imipenem, ceftriaxone, piperacillin/tazobactam). Amplification reaction for detection of *bla*_*TEM*_, *bla*_*SHV*_ and *bla*_*CTX−M*_ genes using triplex PCR was done under the following conditions: initial denaturation at 94 °C for 5 min, followed by 35 cycles of denaturation at 94 °C for 30 s, annealing at 60 °C for 30 s with an extension at 72 °C for 50 s, and a final extension for one cycle at 72 °C for 5 min [28]. In addition, a triplex PCR was done for detection of *bla*_*OXA−48*_, *bla*_*KPC*_ and *bla*_*NDM*_ as well as a duplex PCR was used for detection of *bla*_*IMP*_ *and bla*_*VIM*_ using the following conditions: 10 min at 94 °C and 36 cycles of amplification consisting of 30 s at 94 °C, 40 s at 52 °C, and 50 s at 72 °C, with 5 min at 72 °C for the final extension [29]. The list of primers and the detectable genes are shown in Table 1.

**Table 1:** List of primers used in the detection of beta-lactamase genes and their product sizes

Targeted Gene	Primer Sequence (5′–3′)	Amplicon	References
*bla* *SHV*	F: CGCCTGTGTATTATCTCCCT R: CGAGTAGTCCACCAGATCCT	294 bp	[28]
*bla* *TEM*	F: TTTCGTGTCGCCCTTATTCC R: ATCGTTGTCAGAAGTAAGTTG	404 bp	[28]
*bla* *CTX−M*	F: CGCTGTTGTTAGGAAGTGTG R: GGCTGGGTGAAGTAAGTGA	754 bp	[28]
*bla* *IMP*	F: GGAATAGAGTGGCTTAAYTCTC R: GGTTTAAYAAAACAACCACC	232 bp	[29]
*bla* *NDM*	F: GGTTTGGCGATCTGGTTTTC R: CGGAATGGCTCATCACGATC	621 bp	[29]
*bla* *VIM*	F: GATGGTGTTTGGTCGCATA R: CGAATGCGCAGCACCAG	390 bp	[29]
*bla* *OXA−48*	F: GCGTGGTTAAGGATGAACAC R: CATCAAGTTCAACCCAACCG	438 bp	[29]
*bla* *KPC*	F: CGTCTAGTTCTGCTGTCTTG R: CTTGTCATCCTTGTTAGGCG	232 bp	[29]

### Phylogenetic analysis

Triplex PCR was carried out for detection of TspE4.C2 fragment and *yjaA*,* chuA* genes using the following conditions: denaturation for 4 min at 94 °C, 30 cycles of 5 s at 94 °C and 10 s at 59 °C, and 30 s at 72 °C and a final extension step of 5 min at 72 °C 11 [16]. Primers and corresponding product sizes are illustrated in Table 2.

**Table 2:** List of primers for phylogenetic analysis and their corresponding product sizes

Targeted Gene	Primer sequence (5′–3′)	Amplicon		Reference
*ChuA*	F: GACGAACCA ACGGTCAGGAT R: TGCCGCCAGTACC AAAGACA	279 bp	[16]	
*YjaA*	F: TGAAGTGTCAGGAGACGCT G R: ATGGAGAATGCGTTCCTCAAC	211 bp	[16]	
TspE4.C2	F: GAGTAATGTCGGGGCATTCA R: CGCGCCAACAAAGTATTACG	152 bp	[16]	

### Typing of diarrheagenic *E. coli*

Two multiplex PCR reactions were used for the typing of diarrheagenic *E. coli* using the primers mentioned in Table 3. As follows: assay 1 for detection of *bfpA*, *eae*, and the target of *CVD432*, assay 2 for detection of *LT*, *ST*, *stx1*,* stx2*, and *ipaH*. Assay 1 was performed under the following conditions: 50 °C (2 min, 1 cycle); 95 °C (5 min, 1 cycle); 40 cycles of 95 °C (40 s), 58 °C (1 min), and 72 °C (2 min); and a final extension step at 72 °C (7 min, 1 cycle) [18]. For assay 2 the conditions were: 50 °C (2 min, 1 cycle); 95 °C (5 min, 1 cycle); 40 cycles of 95 °C (45 s), 50 °C (1 min), and 72 °C (1 min); and 72 °C (7 min, 1 cycle) [18].

**Table 3:** PCR primers used in the typing of diarrheagenic *E. coli* and their product sizes

Targeted Gene	Primer sequence (5′–3′)	Amplicon	Reference
*Eae*	F: CTGAACGGCGATTACGCGAA R: CCAGACGATACGATCCAG	917 bp	[18]
*BfpA*	F: AATGGTGCTTGCGCTTGCTGC R: GCCGCTTTATCCAACCTGGTA	326 bp	[18]
*CVD432*	F: CTGGCGAAAGACTGTATCAT R: CAATGTATAGAAATCCGCTGTT	630 bp	[18]
*LT*	F: GGCGACAGATTATACCGTGC R: CGGTCTCTATATTCCCTGTT	450 bp	[18]
*ST*	F: ATTTTTMTTTCTGTATTRTCTT R: CACCCGGTACARGCAGGATT	190 bp	[18]
*IpaH*	F: GTTCCTTGACCGCCTTTCCGATACCGTC R: GCCGGTCAGCCACCCTCTGAGAGTAC	600 bp	[18]
*stx1*	F: ATAAATCGCCATTCGTTGACTAC R: AGAACGCCCACTGAGATCATC	180 bp	[18]
*stx2*	F: GGCACTGTCTGAAACTGCTCC R: TCGCCAGTTATCTGACATTCTG	255 bp	[18]

Electrophoresis in a 1.5% agarose gel (Cleaver Scientific, UK) stained with ethidium bromide (Blutruve, Giza, Egypt) was used to analyze all of the PCR products in our work. The size of PCR amplicons was compared to a 10 kb DNA ladder (SolGent, Korea). Then gel documentation was done using (FAS-DIGI PRO, Germany) and (InGenius, UK) instruments.

### Statistical analysis

Data entry and analysis were done using the Statistical Package for Social Science (IBM SPSS Statistics version 20). Microsoft Office Excel 2023 was used for graphics creation. Qualitative data were presented by frequency distribution (number and percentage) and compared between groups by chi-square tests using SPSS.

## Results

### Prevalence of *E. coli* strains in different isolation sources

**Fig. 2:**
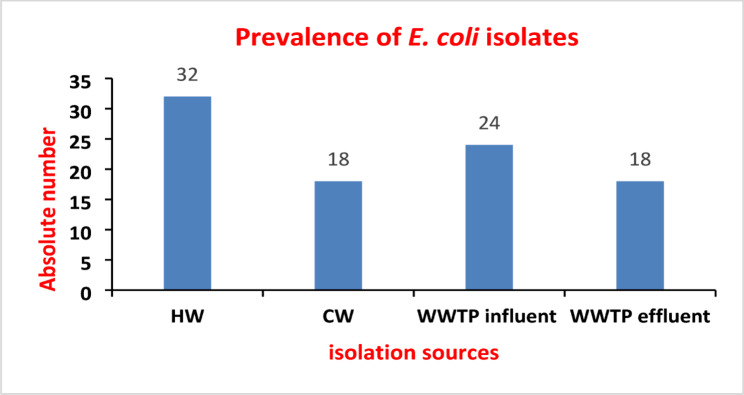
Number of *E. coli* isolates recovered from different wastewater sources

From a total of 120 wastewater samples, 302 bacterial isolates were identified (Table 1 in the supplementary file shows the prevalence of the isolated bacterial strains among different isolation sources). Among these, *E. coli* was the most prevalent species, with 92 (30.5%) isolates detected. The distribution of *E. coli* isolates was as follows: hospital wastewater (*n* = 32), community wastewater (*n* = 18), WWTP influent (*n* = 24), and WWTP effluent (*n* = 18), as illustrated in Fig. 2.

### Antibiotic resistance of total *E. coli* strains

**Fig. 3:**
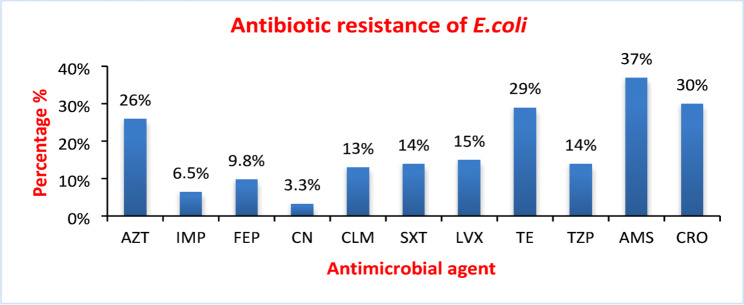
Percentage of *E. coli* isolates resistant to tested antimicrobial agents. Aztreonam (AZT), Imipenem (IMP), Cefepime (FEP), Gentamycin (CN), Chloramphenicol (CLM), Trimethoprim/Sulphamethoxazole (SXT), Levofloxacin (LVX), Tetracycline (TE), Piperacillin/Tazobactam (TZP), Ampicillin/Sulbactam (AMS), Ceftriaxone (CRO)

In our study, 66.3% (61/92) of *E. coli* isolates showed resistance to at least one of the tested antibiotics (Table 2 in the supplementary file shows antibiotic resistance profiles of *E. coli* isolates). WWTPs influent (83.3%, 20/24) was the most common source containing resistant strain, followed by HW (75%, 24/32), CW (50%, 9/18), and WWTPs effluent (44.4%, 8/18). The most frequently detected antibiotics to which the isolates showed resistance were ampicillin/sulbactam (37%, 34/92), followed by ceftriaxone (30.4%, 28/92) and tetracycline (29.3%, 27/92), while gentamycin (3.3%, 3/92) was the most effective antibiotic on the tested strains, followed by imipenem (6.5%, 6/92) as shown in Fig. 3.

### Antibiotic resistance of *E. coli* strains isolated from hospital wastewater (HW) and community wastewater (CW)

75% (24/32) of *E. coli* strains obtained from HW showed resistance to at least one of the tested antibiotics, while among CW isolates, 50% (9/18) were resistant, as shown in Table 4. The highest resistance among HW strains was observed in tetracycline (46.9%, 15/32), followed by ampicillin/sulbactam (40.6%, 13/32), while the most effective one was gentamycin (3.1%, 1/32). Among CW strains, aztreonam (22.2%, 4/18), followed by ceftriaxone (16.7%, 3/18), were the least effective antibiotics, while cefepime, trimethoprim/sulphamethoxazole, and levofloxacin (0% each) were the most effective antibiotics on these strains. The differences in resistance between the two sources were significant in the case of cefepime (*p* = 0.04), sulphamethoxazole/trimethoprim (*p* = 0.02), levofloxacin (*p* = 0.046), and tetracycline (*p* = 0.011).

**Table 4:** Patterns of antibiotic resistance of *E. coli* strains from hospital (HW) and community (CW) wastewaters

	HW (32 strains)		CW (18 strains)		*p*-value
	No*	%**	No*	%**	
Aztreonam	12	37.5%	4	22.2%	0.52
Imipenem	3	9.4%	1	5.6%	0.88
Cefepime	5	15.6%	0	0.0%	0.04
Gentamycin	1	3.1%	1	5.6%	0.69
Chloramphenicol	5	15.6%	2	11.1%	1
Trimethoprim/Sulphamethoxazole	9	28.1%	0	0.0%	0.02
Levofloxacin	6	18.8%	0	0.0%	0.046
Tetracycline	15	46.9%	2	11.1%	0.011
Piperacillin/Tazobactam	8	25%	2	11.1%	0.29
Ampicillin/Sulbactam	13	40.6%	2	11.1%	0.07
Ceftriaxone	9	28.1%	3	16.7%	0.64

* The absolute number of the strains

**Percentages were correlated to the number of *E. coli* strains in each source

### Antibiotic resistance of *E. coli* strains from WWTP influent and effluent

Among WWTP, Influent and effluent, 83.3% (20/24) and 44.4% (8/18) of *E.coli* strains were resistant, respectively. Influent strains showed the highest resistance to ceftriaxone (58.3%, 14/24) and ampicillin/sulbactam (54.2%, 13/24), while effluent strains to ampicillin/sulbactam (33.3%, 6/18). Although lower resistance was found among WWTP effluent than influent strains, however, effluent strains showed higher percentages of resistance to cefepime (11.1% (2/18) vs. 8.3% (2/24)), piperacillin/tazobactam (11.1% (2/18) vs. 4.2% (1/24)), and imipenem (5.6% (1/18) vs. 4.2% (1/24)), as shown in Table 5. The differences were statistically significant in the case of tetracycline (*p* = 0.008) and ceftriaxone (*p* = 0.003).

**Table 5:** Patterns of antibiotic resistance of *E. coli* strains from WWTPs influent and effluent

	WWTPs influent (24 strains)		WWTPs effluent (18 strains)		*P*-value
	No*	%**	No*	%**	
Aztreonam	5	20.8%	3	16.7%	0.6
Imipenem	1	4.2%	1	5.6%	0.67
Cefepime	2	8.3%	2	11.1%	0.66
Gentamycin	1	4.2%	0	0.0%	0.67
Chloramphenicol	5	20.8%	0	0.0%	0.06
Trimethoprim/Sulphamethoxazole	3	12.5%	1	5.6%	0.62
Levofloxacin	7	29.2%	1	5.6%	0.11
Tetracycline	9	37.5%	1	5.6%	0.008
Piperacillin/Tazobactam	1	4.2%	2	11.1%	0.67
Ampicillin/Sulbactam	13	54.2%	6	33.3%	0.32
Ceftriaxone	14	58.3%	2	11.1%	0.003

* The absolute number of the strains

**Percentages were correlated to the number of *E. coli* strains in each source

### Antibiotic resistance of *E. coli* strains based on access, watch, and reserve (AWaRe) classification

According to the WHO classification [30], among the tested antibiotics, there were five access antibiotics (ampicillin/sulbactam, chloramphenicol, gentamicin, trimethoprim/sulphamethoxazole, and tetracycline); five watch (cefepime, imipenem, levofloxacin, piperacillin/tazobactam, and ceftriaxone); and one reserve antibiotic (aztreonam). A total of 52.2% (48/92), 47.8% (44/92), and 26.1% (24/92) of *E. coli* strains exhibit resistance to at least one of the antibiotics belonging to the access, watch, and reserve categories, respectively, that was statistically significant (*p* = 0.001). A slight difference in resistance was noticed between access and watch categories among all sources; however, resistance to access drugs was the highest, while resistance to reserve drugs was the lowest, with statistical significance only in the case of WWTP influent (*p* = 0.002), as illustrated in Table 6.

**Table 6:** Prevalence of resistant *E. coli* to access, watch, and reserve antibiotic categories among different isolation sources

	Access		Watch		Reserve		*p*-value	
	No*	%**	No*	%**	No*	%**		
HW	20	62.5%	19	59.4%	12	37.5%		0.09
CW	6	33.3%	5	27.8%	4	22.2%		0.76
WWTPs influent	16	66.7%	15	62.5%	5	20.8%		0.002
WWTPs effluent	6	33.3%	5	27.8%	3	16.7%		0.5
Total	48	52.2%	44	47.8%	24	26.1%		0.001

* The absolute number of the strains

**Percentages were correlated to the number of *E. coli* strains in each source

### Multidrug resistance (MDR)

Multi-drug resistance (MDR) was defined as resistance to at least 3 tested antibiotics from different classes. 42.6% (26/61) of resistant *E. coli* strains were multidrug-resistant (MDR). The multidrug-resistant (MDR) isolates exhibited diverse resistance profiles across antibiotic classes. Resistance to four classes was most prevalent (30.8%, 8/26), followed by resistance to three (23.1%, 6/26), five (19.2%, 5/26), seven (15.4%, 4/26), and six classes (11.5%, 3/26). The highest frequency of resistance was observed against beta-lactam combinations (84.6%, 22/26), followed by cephalosporins (69.2%, 18/26), tetracyclines (65.4%, 17/26), and monobactams (61.5%, 16/26) (Table 3 in the supplementary file shows MDR patterns of *E. coli* isolates). 50% (12/24) of HW resistant strains were MDR, representing the most common source, followed by WWTPs influent (45%, 9/20), WWTPs effluent (37.5%, 3/8), and CW (22,2%, 2/9), but this difference was statistically nonsignificant (*p* = 0.53). Although MDR isolates were higher in HW than CW, but this was statistically nonsignificant (*p* = 0.15). Similarly, MDR isolates from WWTPs influent were higher than from effluent, but also this was statistically not significant (*p* = 0.73) as illustrated in Table 7.

**Table 7:** Prevalence of MDR *E. coli* isolates among isolation sources

	MDR		*p*-value
	No*	%**	
HW	12	50%	0.15
CW	2	22.2%	
WWTPs influent	9	45%	0.73
WWTPs effluent	3	37.5%	
Total	26	42.6%	0.53

* The absolute number of the strains

**Percentages were correlated to the number of resistant *E. coli* strains in each source

### Molecular detection of beta-lactamase genes

**Fig. 4:**
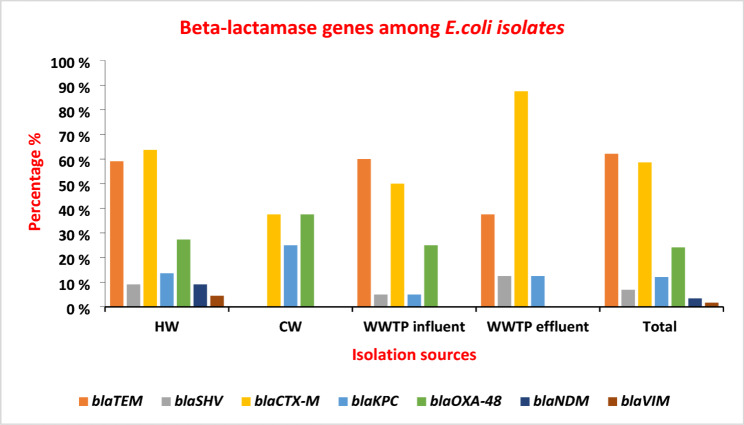
Distribution of beta-lactamase genes among *E. coli* isolates from different sources

A total of 58 *E. coli* strains showed resistance to at least one of the tested beta-lactam antibiotics. These beta-lactam-resistant strains were screened for the presence of ESBLs and carbapenemase genes. Positivity for at least one of the tested genes was detected in 82.8% (48/58) of these strains. *Bla*_*TEM*_ gene (62.1%, 36/58) was the most detected one among extended spectrum beta lactamases, followed by *bla*_*CTX−M*_ (58.6%, 34/58), while the *bla*_*SHV*_ gene was detected in only 6.9% (4/58) of these strains. Among the carbapenemase genes, *bla*_*OXA−48*_ (24.1%, 14/58) was the most detected gene, followed by *bla*_*KPC*_ (12.1%, 7/58), while *bla*_*IMP*_ wasn’t detected in any of the tested strains. The most prevalent beta-lactamase gene among HW (63.7%, 14/22) and WWTPs effluent (87.5%, 7/8) strains was the *bla*_*CTX−M*_ gene. While the *bla*_*TEM*_ gene was the most predominant among CW (100%, 8/8) and WWTP influent (60% 12/20). The differences in prevalence of genes among different sources were statistically significant in the case of *bla*_*TEM*_ (*p* = 0.008) and *bla*_*CTX−M*_ (*p* = 0.025) genes Table 8; Fig. 4.

**Table 8:** Prevalence of ESBL and carbapenemase genes in beta-lactam-resistant *E. coli* strains in different sources

	HW (22 strains)		CW (8 strains)		WWTPs influent (20 strains)		WWTPs effluent (8 strains)		Total		*p*-value
	No*	%**	No*	%**	No*	%**	No*	%**	No*	%**	
*bla* *TEM*	13	59.1%	8	`100%	12	60%	3	37.5%	36	62.1%	0.008
*bla* *SHV*	2	9.1%	0	0%	1	5%	1	12.5%	4	6.9%	0.26
*bla* *CTX−M*	14	63.7%	3	37.5%	10	50%	7	87.5%	34	58.6%	0.025
*bla* *KPC*	3	13.6%	2	25%	1	5%	1	12.5%	7	12.1%	0.13
*bla* *OXA−48*	6	27.3%	3	37.5%	5	25%	0	0%	14	24.1%	0.06
*bla* *NDM*	2	9.1%	0	0%	0	0%	0	0%	2	3.4%	0.066
*bla* *VIM*	1	4.5%	0	0%	0	0%	0	0%	1	1.7%	0.2

* The absolute number of the strains

**Percentage was correlated to number of *E. coli* strains resistant to beta lactam antibiotics in each source

### Phylogenetic classification

It was shown from phylogenetic analysis that most of the *E.coli* strains isolated from different sources were commensals (83.69%, 77/92) while the pathogenic strains were only 16.26% (15/29). The most predominant group among all *E. coli* isolates was group A, followed by group B1 and D, while group B2 was the least detected one. The results were statistically significant (*p* = 0.006) and there was a highly strong negative correlation between the prevalence of commensal groups versus pathogenic groups (Group A and B1 vs. Group B2 and D) in different sources, a correlation coefficient = −1 and p-value < 0.0001 illustrated in Table 9.

**Table 9:** Prevalence of the phylogenetic groups of *E.coli* strains among different sources

	**Commensal strain** **(Group A + Group B1)**		**Pathogenic strain** **(Group B2+Group D)**		**p-value**	**Correlation coefficient (p-value)**
	No*	%**	No*	%**		
HW	15 A+ 11 B1	81.26 %	1 B2+ 5 D	18.76%		
CW	12 A+ 5 B1	94.45 %	1 D	5.56%	0.006	-1 (<0.0001)
WWTPS influent	9 A+ 12 B1	87.5%	1 B2+ 2 D	12.5%		
WWTPS effluent	11 A+ 2 B1	72.22 %	1 B2+ 4 D	27.78 %		
Total	47 A+ 30 B1	83.69 %	3 B2+ 12D	16.26%		

**Percentages were correlated to the number of *E. coli* strains in each source

* The absolute number of the strains

### Typing of diarrheagenic *E. coli*

Out of 92 *E.coli* isolates, only 4 (4.3%) isolates were enterotoxigenic. Among these isolates, one encoded both *ST* and *LT* genes (1%, 1/92) while the other isolates encoded the *ST* gene only (3.26%, 3/29).

## Discussion

Similar to healthcare facilities, wastewater is believed to play a role in the spread of resistant bacteria and their transmission to humans. One of the most prevalent pathogens in wastewater is *E. coli.* In this study, 92 *E.coli* strains were found among 120 wastewater specimens collected from hospitals, community, WWTPs influents and effluents. *E.coli* strains were the most prevalent isolates identified among the collected samples in our study. Similarly, 90 *E.coli* strains were recovered from 288 samples from hospital wastewater in Accra, Ghana, representing the most common isolate found [31]. In addition, *E. coli* was reported as the most prevalent pathogen identified from wastewater samples in the study of Galarde-López et al. [32] and Seguni et al. [33]. The frequent presence of *E. coli* strains in wastewater specimens can be predicted due to the fact that it is a bacterial species found in the microbiota of healthy and sick humans’ digestive tracts, as well as its wide distribution in the environment. Furthermore, its diffusion and dissemination in wastewater suggest that antimicrobial-resistant *E. coli* strains could contaminate aquatic systems or bodies of water and reach the population [34].

In our study, we found that 66.3% (61/92) of *E.coli* strains exhibit resistance to at least one of the tested antibiotics. While a relatively lower percentage of resistance was reported by Paulshus et al., where 42% of *E. coli* strains collected from hospital wastewater (HW), community wastewater (CW), and urban treatment plant in Oslo city, were resistant to at least one of the nine tested antibiotics [7]. We found that isolates showed the highest resistance to ampicillin/sulbactam (37%, 34/92), followed by ceftriaxone (30.4%, 28/92), while the lowest resistance of isolates was found to gentamycin (3.3%, 3/92), followed by imipenem (6.5%, 6/92). Similarly, the same rate of resistance was reported in *E.coli* strains isolated from two third-level hospitals in the Mexico City Metropolitan Zone to ampicillin/sulbactam (36.8%), gentamycin (10.5%), and imipenem (5.3%) [32]. Moreover, in the report of Paulshus et al., resistance of *E.coli* isolates to gentamicin was uncommon (3.2%), whereas resistance to ampicillin was most common (31%) [7]. In addition, multiple studies in several countries have demonstrated the same high incidence of resistance to beta-lactams among *E. coli* bacteria of environmental and clinical sources [35, 36].

The high resistance to ampicillin/sulbactam and ceftriaxone observed in our study is due to the fact that the combination of penicillins and beta-lactamase inhibitors (access drugs) is commonly used for both hospital and community-acquired infections, at 24.8% and 29.2% respectively. In addition, the second prescribed antibiotics was third-generation cephalosporins (15.5%) for community-acquired infections and fluoroquinolones (12.8%) for hospital-acquired infections [37]. Increasing resistance to ampicillin, leading to complicating therapy and emphasizing the necessity for cautious antibiotic management to maintain its effectiveness [38]. Moreover, according to WHO, ceftriaxone is classified in the critically important category (highest and priority antimicrobials) [39] and drugs in this category must be used with caution, because an impairment of their efficacy would lead to the difficulty of treating serious diseases in humans as a result of an absence of therapeutic choices [40]. While the reduced resistance to gentamycin may be explained by its availability for parenteral administration only (with the exception of eye drops), making it less desirable for both the doctor and the patient [41]. Also, imipenem’s low resistance rate could be explained by being a watch drug used for hospitalized patients and administered parenterally [41].

In our work, antibiotic resistance was detected among 75% (24/32) and 50% (9/18) of *E. coli* strains from HW and CW, respectively. Similarly, resistance of *E. coli* strains from HW was also higher in the study of Kwak et al., who found in Stockholm that resistance of *E.coli* strains to at least one of the tested antibiotics was detected among 55% of hospital wastewater and 34% of urban wastewater strains [42]. While only 1% was the difference in resistance between HW and CW, according to the study of Paulshus et al., who found that 45% of the HW and 44% of the CW *E.coli* strains were resistant [7].

Moreover, we found that among HW isolates, resistance to tetracycline (46.9%, 15/32) and ampicillin/sulbactam (40.6%, 13/32) was the highest. Similarly, it was found that *E. coli* strains recovered from five hospitals in Dhaka City showed the highest rate of resistance to tetracycline (100%), followed by ampicillin (97.5%) [43]. This high resistance ratio may be expected as both medications are in the access group, and they are active against a wide range of regularly encountered susceptible infections. We found that CW isolates showed the highest resistance to azetronam (22.2%, 4/18). Likewise, it was reported that the resistance percentage of *E.coli* strains isolated from CW in Timisoara city to azetronam was 32.3% [44]. This indicates high usage of azetronam; however, it belongs to the reserve category, which is reserved for treating infections resulting from MDR organisms and should be treated as a “last resort” choice.

In our work, 83.3% (20/24) of WWTPs influent and 44.4% (8/18) of effluent isolates exhibit resistance to at least one of the tested antibiotics. However, WWTP effluent isolates showed higher percentages of resistance than influent isolates to cefepime, piperacillin/tazobactam, and imipenem antibiotics. While the report of Iweriebor et al. included a higher resistance level in bacteria isolated from WWTP effluent than influent [45]. In addition, it was reported that resistance in treated hospital wastewater is higher than in untreated wastewater in the study conducted in North Ethiopia [46].

Regarding WWTP influent isolates in our study, the highest resistance was found to ceftriaxone (58.3%, 14/24), followed by ampicillin/sulbactam (54.2%, 13/24), while effluent isolates showed the highest resistance to ampicillin/sulbactam (33.3%, 6/18). Notably, *E.coli* strains isolated from treated and untreated wastewaters in the study in North Ethiopia showed the highest resistance to ampicillin (100% each) [46].

We noticed in our work that the resistance of *E.coli* strains among all sources to access antibiotics was the highest, followed by the watch and reserve drugs, respectively. Similarly, resistance to access drugs was the highest, followed by watch and reserve drugs among *E.coli* strains isolated from municipal wastewater in Grenada [47]. However, we found that the differences in resistance to access and watch antibiotics among all sources were small, and the percentages of resistance were close to each other. This means rapid increases in watch antibiotic consumption, reflect challenges in antibiotic stewardship and absence of awareness of guidelines set by WHO for monitoring antibiotic consumption, which aim to at least 60% of total antibiotic use is access to antibiotics. Regarding reserve drugs, the necessity of maintaining low resistance levels is critical, underlining the significance of careful control over the use of these drugs.

Multi-drug resistance, a major health threat, was evident among 42.6% (26/61) of isolates, with the highest frequency among HW and WWTP influent. Higher than our finding (> 71%) was reported for MDR *E. coli* isolates obtained from hospital wastewater and WWTP influent and effluent in Milwaukee, Wisconsin, USA, where MDR frequency was the highest among HW and urban influent WW [48]. We found that although MDR *E. coli* isolated from HW was higher compared to those recovered from CW, but was not statistically significant. Similarly, a study conducted in Timisoara City in Romania reported that MDR *E. coli* isolated from HW was higher than CW isolates, but was statistically nonsignificant [44].

In WWTP effluents, MDR *E. coli* strains were slightly less than in influents. Similarly, a study conducted on Dutch surface water and wastewater found that MDR *E. coli* strains from WWTPs’ effluent were slightly reduced than those in influents (20% vs. 27%, respectively) [49]. This means that these resistant strains persist through the treatment procedures in wastewater treatment plants, meaning a risk of spreading to the environment and infecting humans and animals.

Several beta-lactamase genes were detected in our study, including *bla*_*CTX−M*_, *bla*_*TEM*_, *bla*_*SHV*_, *bla*_*KPC*,_ *bla*_*VIM*_, *bla*_*OXA−48*_, and *bla*_*NDM*_. Though *bla*_*IMP*_ was not found among isolates, the resistance to imipenem could be through *bla*_*VIM*_, *bla*_*OXA−48*_, *bla*_*KPC*,_ or *bla*_*NDM*_ genes [50]. HW was the most common source for isolates containing ESBLs and carbapenemase genes. Similarly, Paulshus et al. [7] found that ESBL-producing *E. coli* were most common among HW than CW and WWTPs, while carbapenemase-producing *E. coli* were more common in CW (76.47%) than in HW (68.09%) [7]. The same situation in Poland, where HW isolates were the most common source for ESBL-producing *E. coli* [51]. Additionally, several beta-lactamase genes such as *bla*_*TEM*_, *bla*_*CTX−M*,_ and *bla*_*SHV*_ were detected in municipal and hospital effluents [51, 52]. These reports, as well as ours, demonstrate that these genes can be disseminated through water systems [51–53], as the genes may remain after the death of bacteria [54], reaching various environments and causing the spread of resistance.

Our work reported that groups A and B1 were the most common in wastewater samples. Similarly, in the study conducted on *E. coli* isolated from waste and surface waters, groups A and B1 were reported as the predominant groups in all types of water [55]. While a study conducted on *E.coli* strains isolated from a municipal wastewater treatment plant reported that group D was the most numerous, followed by group A [56]. In our study, about 83.69% (77/92) of isolates were commensals while only 16.26% (15/92) were pathogenic. So, most of beta lactamase-producer isolates were commensals that may transmit these resistance genes via horizontal gene transfer to intestinal pathogens [57]. Secondly, while these opportunistic bacteria are largely innocuous to healthy people, they can cause infection in susceptible people, such as patients in hospitals, older individuals, or neonates. Thirdly, being exposed to beta-lactamase-producing pathogenic *E. coli* strains can lead to a difficult-to-treat infection even in healthy people. The cumulative risk of being exposed to beta-lactamase-producing *E. coli* (and other resistant commensal bacteria) determines the public health impact. However, far higher than our findings was reported in a South Korean study where the pathogenic ESBL-producing *E.coli* collected from a river were 60% [58]. Additionally, a strong negative correlation between commensal (A and B1) and pathogenic (B2 and D) groups was detected in our study, meaning that as the presence of commensal groups increases in wastewater, pathogenic groups decrease. This was similar to the study of Stoppe et al., in which a negative correlation was found between group A (commensal) and B2 (pathogenic) [59].

In our work, four *E.coli* isolates were identified as enterotoxigenic (ETEC), revealing that wastewater acts as a source for diarrheagenic *E. coli* strains. In addition, Mokracka et al. reported that 12 strains were ETEC, in addition to seven STEC and three EPEC, but no EAEC and EIEC strains were found [56]. Furthermore, the study of Omar & Barnard [60] in South African wastewater treatment plants reported the detection of diarrheagenic *E. coli* at a higher rate, where EPEC (2/10), EHEC (5/10), ETEC (8/10), EIEC (1/10), and EAEC (9/10) were all detected, which supports our findings. The detection of diarrheagenic *E. coli* strains in wastewater, and also their ability to enter surface water via multiple routes, increases the global prevalence of diarrheagenic *E. coli* strains in aquatic systems, posing a considerable danger of waterborne diseases. In addition, the efficacy of antibiotics, used to treat infections produced by these diarrheagenic *E. coli* strains, has been questioned because of the advent of resistant strains to most of the first-line antibiotic drugs [61]. Limitations of our study include that it was conducted in a single city, which may limit the generalizability of the results to other areas with different environmental and infrastructural patterns. Moreover, the study did not determine the concentration of antibiotics in wastewater, which might help link resistance levels with environmental selective pressure.

## Conclusions

This work demonstrates the widespread prevalence of antibiotic-resistant E. coli in several wastewater sources, in particular those associated with hospitals and wastewater treatment plants (WWTPs). The high prevalence of MDR and β-lactamase-producing E. coli strains in hospital wastewater and WWTPs influents emphasizes these as potential reservoirs and dangerous hotspots for resistance gene spreading. Despite community wastewater showed lower resistance rates, it still poses a significant public health risk. Additionally, the persistence of resistant strains in WWTP effluent highlights the shortness of current treatment processes in completely alleviate public health. The presence of diarrheagenic E. coli bacteria, although at low prevalence, focus on potential human exposure and infection through environmental pathways. In addition, the predominance of commensal phylogenetic strains among resistant isolates raises attention about their potential role as reservoirs for resistance genes, which can be horizontally transferred to pathogenic bacteria.

These findings show the urgent need for regular control, improved treatment strategies, and effective stewardship, especially for watch and reserve antibiotics as defined by WHO.

### Recommendations

The usefulness of wastewater-based surveillance as a useful, non-invasive method to track the spread of multidrug-resistant E. coli in both community and hospital settings is highlighted by this work. Since resistant strains have been found in treated effluents as well, we advise doing the following: An early warning system for the emergence and spread of clinically relevant resistance genes in the population is provided by the incorporation of wastewater surveillance into national AMR monitoring systems. By implementing cutting-edge tertiary treatment techniques (such as ozonation, UV disinfection, and membrane filtration) that are more successful than traditional chlorination in eliminating antibiotic-resistant bacteria and resistance genes, wastewater treatment infrastructure can be improved, especially at hospital and industrial discharge points. Antimicrobial use in clinical and community settings should be regulated and monitored since wastewater monitoring data can help guide focused antibiotic stewardship initiatives. To gain a better understanding of the dynamics of resistance gene transfer between environmental, clinical, and animal reservoirs, isolates from environmental samples undergo routine molecular characterisation. By combining these tactics, evidence-based public health policy can be informed and the quiet development of antibiotic resistance can be stopped.

## Supplementary Information

Supplementary Material 1

## Data Availability

All data generated or analyzed during this study are included in this article.

## Abbreviations

ARB: Antibiotic-Resistant Bacteria
*Bla*: Beta lactamase
CW: Community wastewater
CLSI: Clinical Laboratory Standards Institute
EIEC: Enteroinvasive *Escherichia coli*
ETEC: Enterotoxigenic *Escherichia coli*
EAEC: Enteroaggregative *Escherichia coli*
EPEC: Enteropathogenic *Escherichia coli*
ESBLs: Extended Spectrum Betalactamases
HW: Hospital Wastewater
LT: Heat Labile Toxin
STEC: Shiga Toxin-producing *Escherichia coli*
ST: Heat Stable Toxin
SPSS: Statistical Package for Social Science
WWTPs: Wastewater Treatment Plants

## References

[1] World Health Organization. Antimicrobial resistance: an emerging water, sanitation and hygiene issue. Brief Note. 2015;14.

[2] Baquero F, Martínez J-L, Cantón R. Antibiotics and antibiotic resistance in water environments. Curr Opin Biotechnol. 2008;19(3):260–5. 18534838 10.1016/j.copbio.2008.05.006

[3] Kemper N. Veterinary antibiotics in the aquatic and terrestrial environment. Ecol Indic [Internet]. 2008;8(1):1–13. Available from: https://www.sciencedirect.com/science/article/pii/S1470160X07000647

[4] Kümmerer K. Antibiotics in the aquatic environment– A review– Part II. Chemosphere [Internet]. 2009;75(4):435–41. Available from: https://www.sciencedirect.com/science/article/pii/S0045653508015099 10.1016/j.chemosphere.2008.12.006 19178931

[5] Beyene H, Redaie G. Assessment of waste stabilization ponds for the treatment of hospital wastewater: the case of Hawassa. Univ Ref Hosp. 2011;15(1):142–50.

[6] Kraemer SA, Ramachandran A, Perron GG. Antibiotic pollution in the environment: from microbial ecology to public policy. Microorganisms. 2019;7(6):180. 31234491 10.3390/microorganisms7060180 PMC6616856

[7] Paulshus E, Kühn I, Möllby R, Colque P, O’Sullivan K, Midtvedt T, et al. Diversity and antibiotic resistance among Escherichia coli populations in hospital and community wastewater compared to wastewater at the receiving urban treatment plant. Water Res. 2019;161:232–41. 31202110 10.1016/j.watres.2019.05.102

[8] Reinthaler FF, Galler H, Feierl G, Haas D, Leitner E, Mascher F, et al. Resistance patterns of Escherichia coli isolated from sewage sludge in comparison with those isolated from human patients in 2000 and 2009. J Water Health. 2013;11(1):13–20. 23428545 10.2166/wh.2012.207

[9] Berendonk TU, Manaia CM, Merlin C, Fatta-Kassinos D, Cytryn E, Walsh F, et al. Tackling antibiotic resistance: the environmental framework. Nat Rev Microbiol. 2015;13(5):310–7. 25817583 10.1038/nrmicro3439

[10] Guo M-T, Yuan Q-B, Yang J. Insights into the amplification of bacterial resistance to erythromycin in activated sludge. Chemosphere. 2015;136:79–85. 25957255 10.1016/j.chemosphere.2015.03.085

[11] Huang J-J, Hu H-Y, Lu S-Q, Li Y, Tang F, Lu Y, et al. Monitoring and evaluation of antibiotic-resistant bacteria at a municipal wastewater treatment plant in China. Environ Int. 2012;42:31–6. 21450343 10.1016/j.envint.2011.03.001

[12] Kim S, Park H, Chandran K. Propensity of activated sludge to amplify or attenuate Tetracycline resistance genes and Tetracycline resistant bacteria: a mathematical modeling approach. Chemosphere. 2010;78(9):1071–7. 20096919 10.1016/j.chemosphere.2009.12.068

[13] Lenart-Boroń AM, Kulik K, Jelonkiewicz E. Antimicrobial resistance and ESBL genes in E. coli isolated in proximity to a sewage treatment plant. J Environ Sci Heal Part A. 2020;55(14):1571–80. 10.1080/10934529.2020.1826774 33030087

[14] Anastasi EM, Matthews B, Stratton HM, Katouli M. Pathogenic Escherichia coli found in sewage treatment plants and environmental waters. Appl Environ Microbiol. 2012;78(16):5536–41. 22660714 10.1128/AEM.00657-12 PMC3406122

[15] Kaper JB, Nataro JP, Mobley HLT. Pathogenic Escherichia coli. Nat Rev Microbiol. 2004;2(2):123–40. 15040260 10.1038/nrmicro818

[16] Bonacorsi P, Clermont O, Bingen E. Rapid and simple determination of the Escherichia coli phylogenetic group. Appl Environ Microbiol. 2000;66(10):4555–8. 10.1128/aem.66.10.4555-4558.2000 PMC92342 11010916

[17] Nataro JP, Kaper JB. Diarrheagenic Escherichia coli. Clin Microbiol Rev. 1998;11(1):142–201. 9457432 10.1128/cmr.11.1.142 PMC121379

[18] Aranda KRS, Scaletsky ICA. Evaluation of multiplex PCRs for diagnosis of infection with diarrheagenic Escherichia coli and Shigella spp. J Clin Microbiol. 2004;42(12):5849–53. 10.1128/JCM.42.12.5849-5853.2004 PMC535216 15583323

[19] Johnson TJ, Logue CM, Johnson JR, Kuskowski MA, Sherwood JS, Barnes HJ, et al. Associations between multidrug resistance, plasmid content, and virulence potential among extraintestinal pathogenic and commensal Escherichia coli from humans and poultry. Foodborne Pathog Dis. 2012;9(1):37–46. 21988401 10.1089/fpd.2011.0961 PMC3250628

[20] Bartlett JG. 2005-6 pocket book of infectious disease therapy. Lippincott Williams & Wilkins; 2005.

[21] Siu L-K. Antibiotics: action and resistance in gram-negative bacteria. J Microbiol Immunol Infect Wei Mian Yu Gan Ran Za Zhi. 2002;35(1):1–11. 11950113

[22] Santosaningsih D, Fadriyana AP, David NI. Prevalence and Abundance of Beta-Lactam Resistance Genes in Hospital Wastewater and Enterobacterales Wastewater Isolates. 2023. 10.3390/tropicalmed8040193 PMC10146145 37104319

[23] Zurfluh K, Hächler H, Nüesch-Inderbinen M, Stephan R. Characteristics of extended-spectrum β-lactamase-and carbapenemase-producing Enterobacteriaceae isolates from rivers and lakes in Switzerland. Appl Environ Microbiol. 2013;79(9):3021–6. 23455339 10.1128/AEM.00054-13 PMC3623138

[24] Queenan AM, Bush K. Carbapenemases: the versatile β-lactamases. Clin Microbiol Rev. 2007;20(3):440–58. 17630334 10.1128/CMR.00001-07 PMC1932750

[25] Hocquet D, Muller A, Bertrand X. What happens in hospitals does not stay in hospitals: antibiotic-resistant bacteria in hospital wastewater systems. J Hosp Infect. 2016;93(4):395–402. 26944903 10.1016/j.jhin.2016.01.010

[26] Hutinel M, Fick J, Larsson DGJ, Flach C-F. Investigating the effects of municipal and hospital wastewaters on horizontal gene transfer. Environ Pollut [Internet]. 2021;276:116733. Available from: https://www.sciencedirect.com/science/article/pii/S0269749121003134 10.1016/j.envpol.2021.116733 33631686

[27] Dashti AA, Jadaon MM, Dashti H. Heat Treatment of Bacteria: A Simple Method of DNA Extraction for Molecular Heat Treatment of Bacteria: A Simple Method of DNA Extraction for Molecular Techniques. 2009;(October 2014).

[28] Hassan MI, Alkharsah KR, Alzahrani AJ, Obeid OE, Khamis AH, Diab A. Detection of extended spectrum beta-lactamases-producing isolates and effect of AmpC overlapping. J Infect Dev Ctries. 2013; 7(8): 618-29. 10.3855/jidc.2919 23949298

[29] Poirel L, Walsh TR, Cuvillier V, Nordmann P. Multiplex PCR for detection of acquired carbapenemase genes. Diagn Microbiol Infect Dis [Internet]. 2011;70(1):119–23. Available from: 10.1016/j.diagmicrobio.2010.12.002 10.1016/j.diagmicrobio.2010.12.002 21398074

[30] Organization WH. The 2019 WHO AWaRe classification of antibiotics for evaluation and monitoring of use. In: The 2019 WHO AWaRe classification of antibiotics for evaluation and monitoring of use. 2019.

[31] Addae-Nuku DS, Kotey FCN, Dayie NTKD, Osei M-M, Tette EMA, Debrah P, et al. Multidrug-resistant bacteria in hospital wastewater of the Korle Bu teaching hospital in accra, Ghana. Environ Health Insights. 2022;16:11786302221130612. 10.1177/11786302221130613 PMC9597020 36311334

[32] Galarde-López M, Velazquez-Meza ME, Bobadilla-del-Valle M, Cornejo-Juárez P, Carrillo-Quiroz BA, Ponce-de-León A, et al. Antimicrobial resistance patterns And clonal distribution of E. coli, Enterobacter spp. And acinetobacter spp. Strains isolated from two hospital wastewater plants. Antibiotics. 2022;11(5):601. 35625245 10.3390/antibiotics11050601 PMC9137823

[33] Seguni NZ, Kimera ZI, Msafiri F, Mgaya FX, Joachim A, Mwingwa A et al. Multidrug-resistant Escherichia coli and Klebsiella pneumoniae isolated from hospital sewage flowing through community sewage system and discharging into the Indian Ocean. Bull Natl Res Cent [Internet]. 2023;47(1). Available from: 10.1186/s42269-023-01039-4

[34] Lamba M, Ahammad SZ. Sewage treatment effluents in delhi: A key contributor of β-lactam resistant bacteria and genes to the environment. Chemosphere. 2017;188:249–56. 28886559 10.1016/j.chemosphere.2017.08.133

[35] Bréchet C, Plantin J, Sauget M, Thouverez M, Talon D, Cholley P, et al. Wastewater treatment plants release large amounts of extended-spectrum β-lactamase–producing Escherichia coli into the environment. Clin Infect Dis. 2014;58(12):1658–65. 24795329 10.1093/cid/ciu190

[36] Bardhan T, Chakraborty M, Bhattacharjee B. Prevalence of colistin-resistant, carbapenem-hydrolyzing Proteobacteria in hospital water bodies and out-falls of West bengal, India. Int J Environ Res Public Health. 2020;17(3). 10.3390/ijerph17031007 PMC7037630 32033408

[37] Versporten A, Zarb P, Caniaux I, Gros M-F, Drapier N, Miller M, et al. Antimicrobial consumption and resistance in adult hospital inpatients in 53 countries: results of an internet-based global point prevalence survey. Lancet Glob Heal. 2018;6(6):e619–29. 10.1016/S2214-109X(18)30186-4 29681513

[38] KaushiK D, Mohan M, BoraDe DM, Swami OC. Ampicillin: rise fall and resurgence. J Clin Diagn Res JCDR. 2014;8(5):ME01. 24995206 10.7860/JCDR/2014/8777.4356 PMC4080027

[39] Organization WH. Critically important antimicrobials for human medicine. In: Critically important antimicrobials for human medicine. 2016.

[40] Resistance WHOAG. on IS of A. Critically important antimicrobials for human medicine. World Health Organization; 2012.

[41] Organization WH. The selection and use of essential medicines: report of the WHO expert committee, 2005 (including the 14th model list of essential Medicines). Volume 933. World Health Organization; 2006. 17566509

[42] Kwak Y-K, Colque P, Byfors S, Giske CG, Möllby R, Kühn I. Surveillance of antimicrobial resistance among Escherichia coli in wastewater in Stockholm during 1 year: does it reflect the resistance trends in the society? Int J Antimicrob Agents. 2015;45(1):25–32. 25465520 10.1016/j.ijantimicag.2014.09.016

[43] SM, MI, SC, MAM. Detection of Tetracycline and ampicillin resistant E. coli and Salmonella species from hospital wastewater. Bangladesh Vet. 2024;40(1–2):16–24.

[44] Gaşpar C-M, Cziszter LT, Lăzărescu CF, Ţibru I, Pentea M, Butnariu M. Antibiotic resistance among Escherichia coli isolates from hospital wastewater compared to community wastewater. Water. 2021;13(23):3449.

[45] Iweriebor BC, Gaqavu S, Obi LC, Nwodo UU, Okoh AI. Antibiotic susceptibilities of Enterococcus species isolated from hospital and domestic wastewater effluents in alice, Eastern cape Province of South Africa. Int J Environ Res Public Health. 2015;12(4):4231–46. 25893999 10.3390/ijerph120404231 PMC4410244

[46] Asfaw T, Negash L, Kahsay A, Weldu Y. Antibiotic resistant bacteria from treated and untreated hospital wastewater at ayder referral hospital, mekelle, North Ethiopia. Adv Microbiol. 2017;07(12):871–86.

[47] Matthew-Bernard M, Farmer-Diaz K, Dolphin-Bond G, Matthew-Belmar V, Cheetham S, Mitchell K et al. Phenotypic antibiotic resistance patterns of Escherichia coli isolates from clinical UTI samples and municipal wastewater in a Grenadian community. Int J Environ Res Public Health. 2025;22(1). 10.3390/ijerph22010097 PMC11765413 39857550

[48] Liedhegner E, Bojar B, Beattie RE, Cahak C, Hristova KR, Skwor T. Similarities in virulence and extended spectrum Beta-Lactamase gene profiles among Cefotaxime-Resistant Escherichia coli wastewater and clinical isolates. Antibiotics. 2022;11(2). 10.3390/antibiotics11020260 PMC8868091 35203862

[49] Blaak H, Lynch G, Italiaander R, Hamidjaja RA, Schets FM, de Roda Husman AM. Multidrug-resistant and extended spectrum beta-lactamase-producing Escherichia coli in Dutch surface water and wastewater. PLoS ONE. 2015;10(6):e0127752. 26030904 10.1371/journal.pone.0127752 PMC4452230

[50] Vijayakumar S, Gopi R, Gunasekaran P, Bharathy M, Walia K, Anandan S, et al. Molecular characterization of invasive carbapenem-resistant acinetobacter baumannii from a tertiary care hospital in South India. Infect Dis Ther. 2016;5:379–87. 27553951 10.1007/s40121-016-0125-y PMC5019981

[51] Korzeniewska E, Korzeniewska A, Harnisz M. Antibiotic resistant Escherichia coli in hospital and municipal sewage and their emission to the environment. Ecotoxicol Environ Saf. 2013;91:96–102. 23433837 10.1016/j.ecoenv.2013.01.014

[52] Adegoke AA, Madu CE, Aiyegoro OA, Stenström TA, Okoh AI. Antibiogram and beta-lactamase genes among cefotaxime resistant E. coli from wastewater treatment plant. Antimicrob Resist Infect Control. 2020;9(1):1–12. 32164766 10.1186/s13756-020-0702-4 PMC7068970

[53] Tacão M, Correia A, Henriques I. Resistance to broad-spectrum antibiotics in aquatic systems: anthropogenic activities modulate the dissemination of Bla CTX-M-like genes. Appl Environ Microbiol. 2012;78(12):4134–40. 22492443 10.1128/AEM.00359-12 PMC3370516

[54] Lood R, Ertürk G, Mattiasson B. Revisiting antibiotic resistance spreading in wastewater treatment plants–bacteriophages as a much neglected potential transmission vehicle. Front Microbiol. 2017;8:2298. 29209304 10.3389/fmicb.2017.02298 PMC5702337

[55] Figueira V, Serra E, Manaia CM. Differential patterns of antimicrobial resistance in population subsets of Escherichia coli isolated from waste- and surface waters. Sci Total Environ [Internet]. 2011;409(6):1017–23. Available from: https://www.sciencedirect.com/science/article/pii/S0048969710013148 10.1016/j.scitotenv.2010.12.011 21215425

[56] Mokracka J, Koczura R, Jabłońska L, Kaznowski A. Phylogenetic groups, virulence genes and quinolone resistance of integron-bearing Escherichia coli strains isolated from a wastewater treatment plant. Antonie Van Leeuwenhoek. Int J Gen Mol Microbiol. 2011;99(4):817–24. 10.1007/s10482-011-9555-4 PMC3079074 21293926

[57] Hunter PR, Wilkinson DC, Catling LA, Barker GC. Meta-analysis of experimental data concerning antimicrobial resistance gene transfer rates during conjugation. Appl Environ Microbiol. 2008;74(19):6085–90. 18708517 10.1128/AEM.01036-08 PMC2565951

[58] Jang J, Suh Y-S, Di DYW, Unno T, Sadowsky MJ, Hur H-G. Pathogenic Escherichia coli strains producing extended-spectrum β-lactamases in the Yeongsan river basin of South Korea. Environ Sci Technol. 2013;47(2):1128–36. 23256438 10.1021/es303577u

[59] Stoppe N, de Silva C, Carlos JS, Sato C, Saraiva MIZ, Ottoboni AM et al. LMM,. Worldwide phylogenetic group patterns of Escherichia coli from commensal human and wastewater treatment plant isolates. Front Microbiol. 2017;8(DEC). 10.3389/fmicb.2017.02512 PMC5742620 29312213

[60] Omar KB, Barnard TG. The occurrence of pathogenic Escherichia coli in South African wastewater treatment plants as detected by multiplex PCR. Water SA. 2010;36(2):172–6.

[61] Ishii S, Sadowsky MJ. Escherichia coli in the environment: implications for water quality and human health. Microbes Environ. 2008;23(2):101–8. 21558695 10.1264/jsme2.23.101

