# Supplementary material for: Multidrug-resistant E. Coli in wastewater sources: a comparative study and identification of resistance hotspots
Source: BMC Microbiol. 2025 Aug 12;25:498. doi: 10.1186/s12866-025-04244-5 (PMC12341253; doi:10.1186/s12866-025-04244-5)
Supplement: Supplementary file 1 — Supplementary Material 1 [file 12866_2025_4244_MOESM1_ESM.docx]

**Supplementary data**

**Table 1****.** Prevalence of the isolated bacterial strains among different isolation sources.

|  | **HW**  **(107 isolates (** | | **CW**  **(64 isolates)** | | **WWTP influent**  **(76 isolates)** | | **WWTP effluent**  **(55 isolates)** | |
| --- | --- | --- | --- | --- | --- | --- | --- | --- |
|  | No. | % * | No. | % * | No. | (% * | No. | % * |
| *Escherichia coli* | 32 | 29.9% | 18 | 28.1% | 24 | 31.6% | 18 | 32.7% |
| *Klebsiella pneumoniae* | 31 | 29% | 16 | 25% | 20 | 26.3% | 14 | 25.5% |
| *Staphylococcus aureus* | 18 | 16.8% | 15 | 23.4% | 15 | 19.7% | 5 | 9.1% |
| *Coagulase-negative staphylococci* | 7 | 6.5% | 3 | 4.7% | 1 | 1.3% | 1 | 1.8% |
| *Pseudomonas aeruginosa* | 1 | 0.9% | 2 | 3.1% | 6 | 7.9% | 13 | 23.6% |
| *Proteus vulgaris* | 2 | 1.9% | 2 | 3.1% | 6 | 7.9% | 0 | 0.0% |
| *Proteus mirabilis* | 3 | 2.8% | 2 | 3.1% | 0 | 0.0% | 0 | 0.0% |
| *Enterobacter spp.* | 1 | 0.9% | 2 | 3.1% | 1 | 1.3% | 1 | 1.8% |
| *Citrobacter spp.* | 4 | 3.7% | 0 | 0.0% | 1 | 1.3% | 0 | 0.0% |
| *Shigella spp.* | 5 | 4.7% | 2 | 3.1% | 2 | 2.6% | 2 | 3.6% |
| *Salmonella spp.* | 2 | 1.9% | 1 | 1.6% | 0 | 0.0% | 0 | 0.0% |
| *Acinetobacter spp.* | 1 | 0.9% | 1 | 1.6% | 0 | 0.0% | 1 | 1.8% |

*Percentages were correlated to the number of isolates in each source

**Table 2.** Antibiotic Resistance Profiles of *E. coli* Isolates

| **Isolate** | **Source** | **AZT** | **IMP** | **FEP** | **CN** | **CLM** | **SXT** | **LVX** | **TE** | **TPZ** | **AMS** | **CRO** |
| --- | --- | --- | --- | --- | --- | --- | --- | --- | --- | --- | --- | --- |
| E1 | Hospital | R | I | R | S | S | R | R | S | S | S | S |
| E2 | Hospital | R | S | R | I | S | R | R | S | S | S | S |
| E3 | Hospital | R | S | I | S | S | S | S | S | S | S | S |
| E4 | Community | S | S | S | R | S | S | S | S | S | S | S |
| E5 | WWTP effluent | R | S | R | S | S | S | S | S | S | S | S |
| E6 | WWTP effluent | R | R | R | I | S | S | S | S | S | R | R |
| E7 | Community | R | S | I | S | S | S | S | S | S | R | R |
| E8 | Community | I | S | I | S | S | S | S | S | S | S | S |
| E9 | Hospital | R | S | S | S | S | S | S | R | R | R | S |
| E10 | Hospital | R | R | R | S | R | R | I | R | R | R | S |
| E11 | WWTP influent | S | S | S | S | S | S | S | R | S | R | S |
| E12 | Hospital | R | S | S | S | S | R | S | R | S | R | S |
| E13 | Hospital | S | I | S | S | S | S | I | R | R | R | S |
| E14 | WWTP effluent | R | S | S | S | S | S | S | R | S | R | S |
| E15 | Hospital | R | I | R | S | S | R | I | R | R | R | S |
| E16 | Hospital | S | S | S | S | S | R | R | R | R | R | R |
| E17 | WWTP influent | S | S | S | R | R | R | R | R | S | R | R |
| E18 | WWTP effluent | S | S | S | S | S | R | R | S | I | R | R |
| E19 | Hospital | S | S | S | S | S | S | S | S | S | S | I |
| E20 | Hospital | S | S | S | S | S | S | S | R | S | S | I |
| E21 | Hospital | S | S | S | S | S | S | S | S | S | S | S |
| E22 | Hospital | S | I | S | S | S | S | S | S | S | S | R |
| E23 | Hospital | S | S | S | S | S | S | S | S | S | S | S |
| E24 | Hospital | S | S | S | S | S | S | S | S | S | S | S |
| E25 | Community | S | S | S | S | S | S | S | S | S | S | I |
| E26 | Community | S | S | S | S | R | S | S | I | R | I | S |
| E27 | Community | S | S | S | S | S | S | S | S | S | S | S |
| E28 | Community | S | S | S | S | S | S | S | S | S | S | S |
| E29 | Community | S | S | S | S | S | S | S | I | S | S | S |
| E30 | Community | S | R | S | S | S | S | S | S | S | S | S |
| E31 | Community | S | S | S | S | S | S | S | S | R | S | S |
| E32 | Community | S | S | S | S | S | S | S | S | S | S | S |
| E33 | Hospital | S | S | S | S | S | S | S | S | S | S | S |
| E34 | Hospital | S | S | S | S | S | S | S | S | S | S | S |
| E35 | Hospital | S | S | S | S | S | S | S | R | S | I | S |
| E36 | Hospital | S | S | S | S | S | S | S | S | S | I | S |
| E37 | Community | S | I | S | S | S | S | S | S | S | S | R |
| E38 | WWTP influent | R | S | R | S | S | R | R | R | I | R | S |
| E39 | WWTP influent | S | S | S | S | S | S | S | S | S | S | S |
| E40 | WWTP influent | R | S | S | S | S | R | S | S | S | S | S |
| E41 | WWTP effluent | S | S | S | S | S | S | S | S | S | S | S |
| E42 | WWTP effluent | S | S | S | S | S | S | S | S | S | S | S |
| E43 | WWTP effluent | S | S | S | S | S | S | S | S | S | S | S |
| E44 | WWTP effluent | S | S | S | S | S | S | S | S | S | S | S |
| E45 | WWTP effluent | S | S | S | S | S | S | S | S | S | S | S |
| E46 | WWTP effluent | S | S | S | S | S | S | S | S | S | S | S |
| E47 | Community | R | S | S | S | S | S | S | S | S | S | S |
| E48 | Community | S | S | S | S | S | S | S | I | S | S | S |
| E49 | Community | R | S | S | S | S | S | S | R | S | I | S |
| E50 | Community | R | S | S | S | S | S | S | R | S | S | S |
| E51 | Hospital | R | R | S | R | S | R | R | S | R | R | S |
| E52 | Hospital | R | R | S | S | R | R | R | R | R | R | S |
| E53 | Hospital | R | S | S | S | R | R | R | R | R | R | S |
| E54 | WWTP influent | S | S | S | S | S | S | S | R | S | R | R |
| E55 | WWTP influent | S | S | S | S | S | S | S | S | S | I | R |
| E56 | WWTP influent | S | S | S | S | S | S | S | R | S | R | R |
| E57 | WWTP influent | R | R | I | S | S | S | R | R | R | R | R |
| E58 | WWTP influent | S | S | S | S | R | S | R | R | S | R | R |
| E59 | WWTP influent | I | S | S | S | R | S | S | R | S | R | R |
| E60 | WWTP influent | R | I | R | I | S | S | R | R | S | R | R |
| E61 | WWTP influent | I | S | S | S | R | S | R | I | S | I | R |
| E62 | WWTP influent | R | S | S | S | R | S | R | I | S | S | R |
| E63 | WWTP influent | S | S | S | S | S | S | S | S | S | S | S |
| E64 | WWTP influent | S | S | S | S | S | S | S | S | S | S | S |
| E65 | WWTP influent | S | S | S | S | S | S | S | I | S | S | R |
| E66 | WWTP influent | S | S | S | S | S | S | S | S | S | R | S |
| E67 | WWTP influent | I | S | S | S | S | S | S | S | S | S | R |
| E68 | WWTP influent | S | S | S | S | S | S | S | S | S | S | S |
| E69 | WWTP influent | S | S | S | S | S | S | S | S | S | R | S |
| E70 | Hospital | S | S | S | S | S | S | S | R | S | I | R |
| E71 | Hospital | I | S | S | S | S | S | S | R | S | R | S |
| E72 | Hospital | S | S | S | S | S | S | S | S | S | R | S |
| E73 | Hospital | S | S | S | S | S | S | S | S | S | S | R |
| E74 | Hospital | S | S | S | S | S | S | S | R | S | S | R |
| E75 | Hospital | R | S | S | S | R | S | S | R | S | R | R |
| E76 | Hospital | S | S | S | S | R | S | S | I | S | S | R |
| E77 | Hospital | R | S | S | S | S | S | S | R | S | R | R |
| E78 | Hospital | S | S | S | S | S | S | S | S | S | S | R |
| E79 | WWTP effluent | S | S | S | S | S | S | S | S | R | R | S |
| E80 | WWTP effluent | S | S | S | S | S | S | S | S | R | S | S |
| E81 | WWTP effluent | S | S | S | S | S | S | S | S | S | R | S |
| E82 | WWTP effluent | S | S | S | S | S | S | S | S | S | S | S |
| E83 | WWTP effluent | S | S | S | S | S | S | S | S | S | R | S |
| E84 | Community | S | S | S | S | S | S | S | S | S | S | S |
| E85 | WWTP effluent | I | S | S | S | S | S | S | S | S | S | S |
| E86 | Hospital | I | S | S | S | S | S | S | S | S | I | S |
| E87 | WWTP effluent | I | S | S | S | S | S | S | S | S | S | S |
| E88 | Community | S | R | S | S | R | S | S | I | S | R | R |
| E89 | WWTP effluent | S | S | S | S | S | S | S | S | S | I | S |
| E90 | WWTP influent | S | S | S | S | S | S | S | S | S | R | S |
| E91 | WWTP influent | I | S | S | S | S | S | S | S | S | R | R |
| E92 | WWTP influent | I | S | S | S | S | S | S | S | S | S | R |

**R** = Resistant, **S** = Sensitive, **I** = Intermediate
**AZT** = Aztreonam, **IMP** = Imipenem, **FEP** = Cefepime, **CN** = Gentamycin, **CLM** = Chloramphenicol, **SXT** = Trimethoprim/Sulphamethoxazole, **LVX** = Levofloxacin, **TE** = Tetracycline, **TZP** = Piperacillin/Tazobactam, **AMS** = Ampicillin/Sulbactam, **CRO** = Ceftriaxone.

**Table 3.** MDR patterns of *E. coli* isolates

| **MDR pattern** | **Source** |
| --- | --- |
| AZT, FEP, SXT, LVX* | Hospital |
| AZT, IMP, FEP, AMS, CRO | WWTP effluent |
| AZT, AMS, CRO | Community |
| AZT, TE, TPZ, AMS | Hospital |
| AZT, IMP, FEP, CLM, SXT, TE, TPZ, AMS | Hospital |
| AZT, SXT, TE, AMS | Hospital |
| AZT, TE, AMS | WWTP effluent |
| AZT, FEP, SXT, TE, TPZ, AMS | Hospital |
| SXT, LVX, TE, TPZ, AMS, CRO | Hospital |
| CN, CLM, SXT, LVX, TE, AMS, CRO | WWTP influent |
| SXT, LVX, AMS, CRO | WWTP effluent |
| AZT, FEP, SXT, LVX, TE, AMS | WWTP influent |
| AZT, IMP, CN, SXT, LVX, TE, TPZ, AMS | Hospital |
| AZT, IMP, CLM, SXT, LVX, TE, TPZ, AMS | Hospital |
| TE, AMS, CRO* | Hospital, WWTP influent |
| AZT, IMP, LVX, TE, AMS, CRO | WWTP influent |
| CLM, LVX, TE, AMS, CRO | WWTP influent |
| CLM, TE, AMS, CRO | WWTP influent |
| AZT, FEP, LVX, TE, AMS, CRO | WWTP influent |
| CLM, LVX, CRO | WWTP influent |
| AZT, CLM, LVX, CRO | WWTP influent |
| AZT, CLM, TE, AMS, CRO | Hospital |
| AZT, TE, AMS, CRO | Hospital |
| IMP, CLM, AMS, CRO | Community |

*Two isolates had this resistance pattern

**AZT** = Aztreonam, **IMP** = Imipenem, **FEP** = Cefepime, **CN** = Gentamycin, **CLM** = Chloramphenicol, **SXT** = Trimethoprim/Sulphamethoxazole, **LVX** = Levofloxacin, **TE** = Tetracycline, **TZP** = Piperacillin/Tazobactam, **AMS** = Ampicillin/Sulbactam, **CRO** = Ceftriaxone.


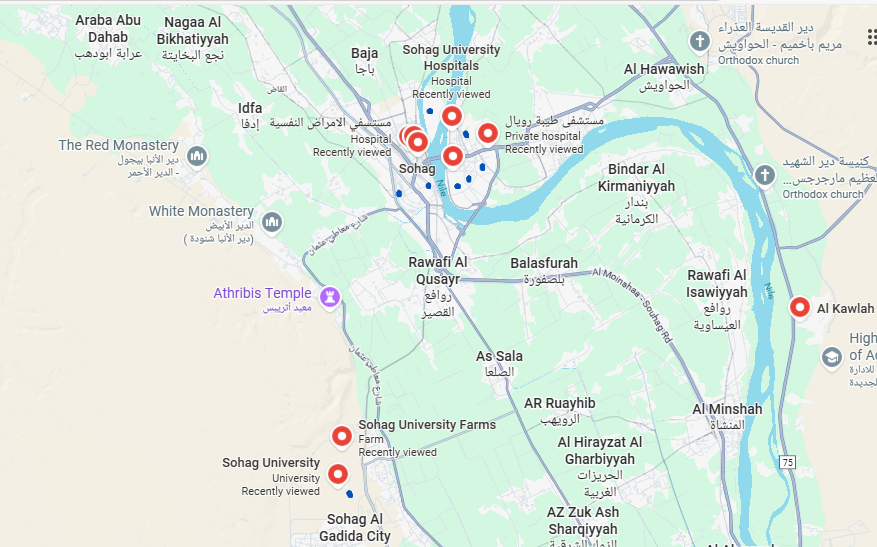


**Fig 1.** Map showing the sampling locations in Sohag City, Egypt. Red markers indicate Sohag University Hospital, Masr Hospital, Tiba Royal Hospital, Sohag University, and Al Kawlah Wastewater Treatment Plant (WWTP). Blue markers represent approximate residential sites and student dormitory, where community wastewater samples were collected.


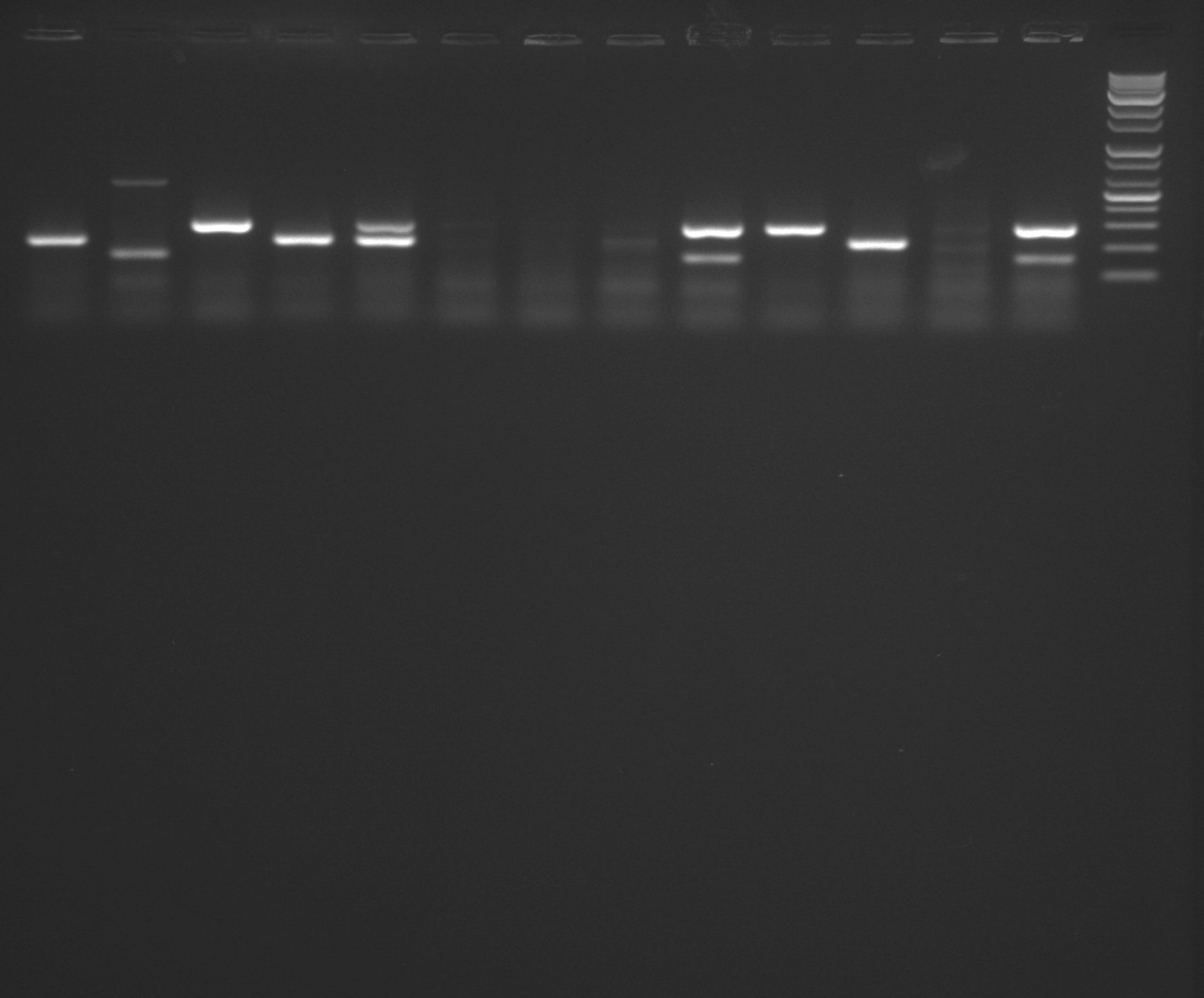


**13 12 11 10 9 8 7 6 5 4 3 2 1 M**

***ChuA***

**211 bp**

**279 bp**

***YjaA***

**TspE4.C2**

**152 bp**

**Fig 2. Triplex PCR profiles specific for *chuA* and *yjaA* genes and DNA fragment TSPE4.C2. Lane M: 10 kb molecular weight marker; lane 1, 5: *ChuA*  (279 bp) and *TspE4.C2* (152 bp); lane 3,10,13: *YjaA* (200 bp); lane 4,11: *ChuA* (279 bp); lane 9: *ChuA* (279 bp) and *YjaA* (211 BP).**

**
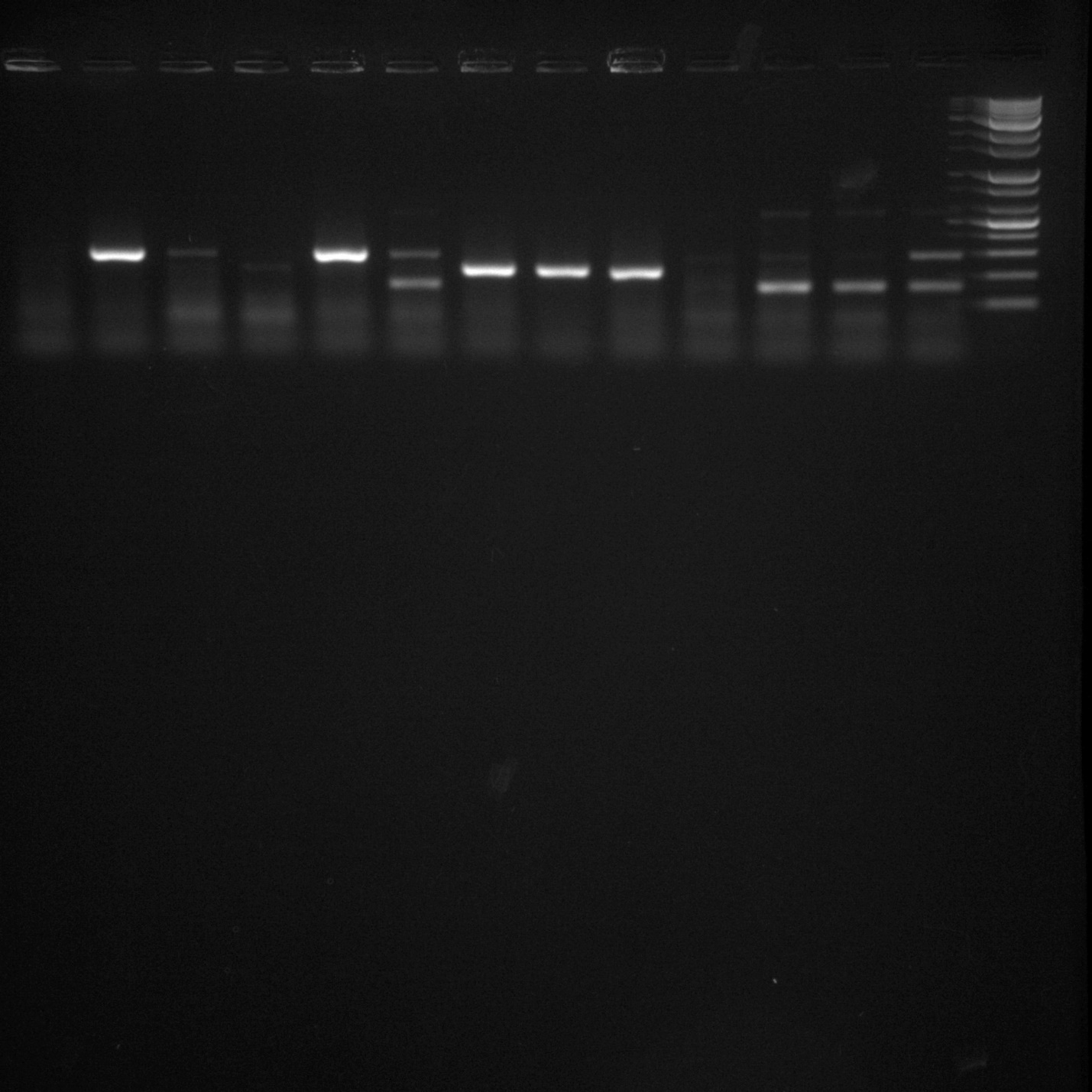
**

***ChuA***

***YjaA***

TspE4C2

***TspE4C2***

**279 bp**

200 bp

**211 bp**

**152 bp**

13 12 11 10 9 8 7 6 5 4 3 2 1 M

**Fig 3. Triplex PCR profiles specific for *chuA* and *yjaA* genes and DNA fragment *TSPE4.C2*. Lane M: 10 kb molecular weight marker; lane 1, 8: *ChuA*  (279 bp) and** ***TspE4.C2* (152 bp); lane 2, 3: *TspE4.C2* (152 bp); lane 5,6,7: *YjaA* (211 bp); lane 9, 11, 12: *ChuA* (279 bp).**


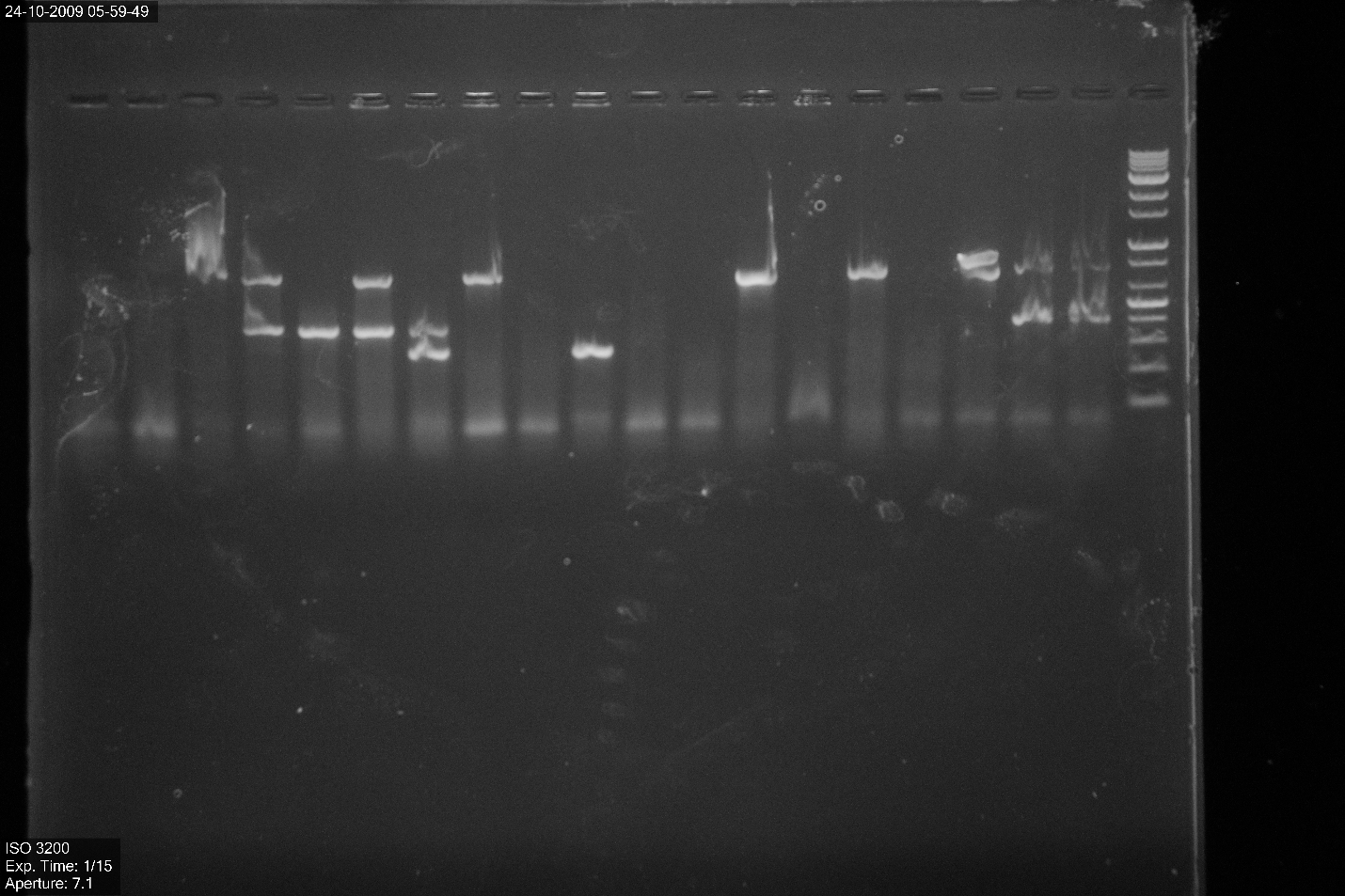


19 18 17 16 15 14 13 12 11 10 9 8 7 6 5 4 3 2 1 M

***bla_TEM_***

***bla_SHV_***

***bla_CTX-M_***

**404 bp**

294 bp

**294 bp**

**754 bp**

**Fig 4. Triplex PCR profiles specific for** ***bla_TEM_*,** ***bla_SHV,_* and** ***bla_CTX-M_*. Lane M: 10 kb molecular weight marker; lane 1,2,14,16: *bla_CTX-M_* (754 bp) and *bla_TEM_* (404 bp); lane 3,5,7,12: *bla_CTX-M_* (754 bp); lane 10: *bla_SHV_* (294 bp); lane 13: *bla_TEM_* (404 bp) and *bla_SHV_* (294 bp); lane 15: *bla_TEM_* (404 bp).**


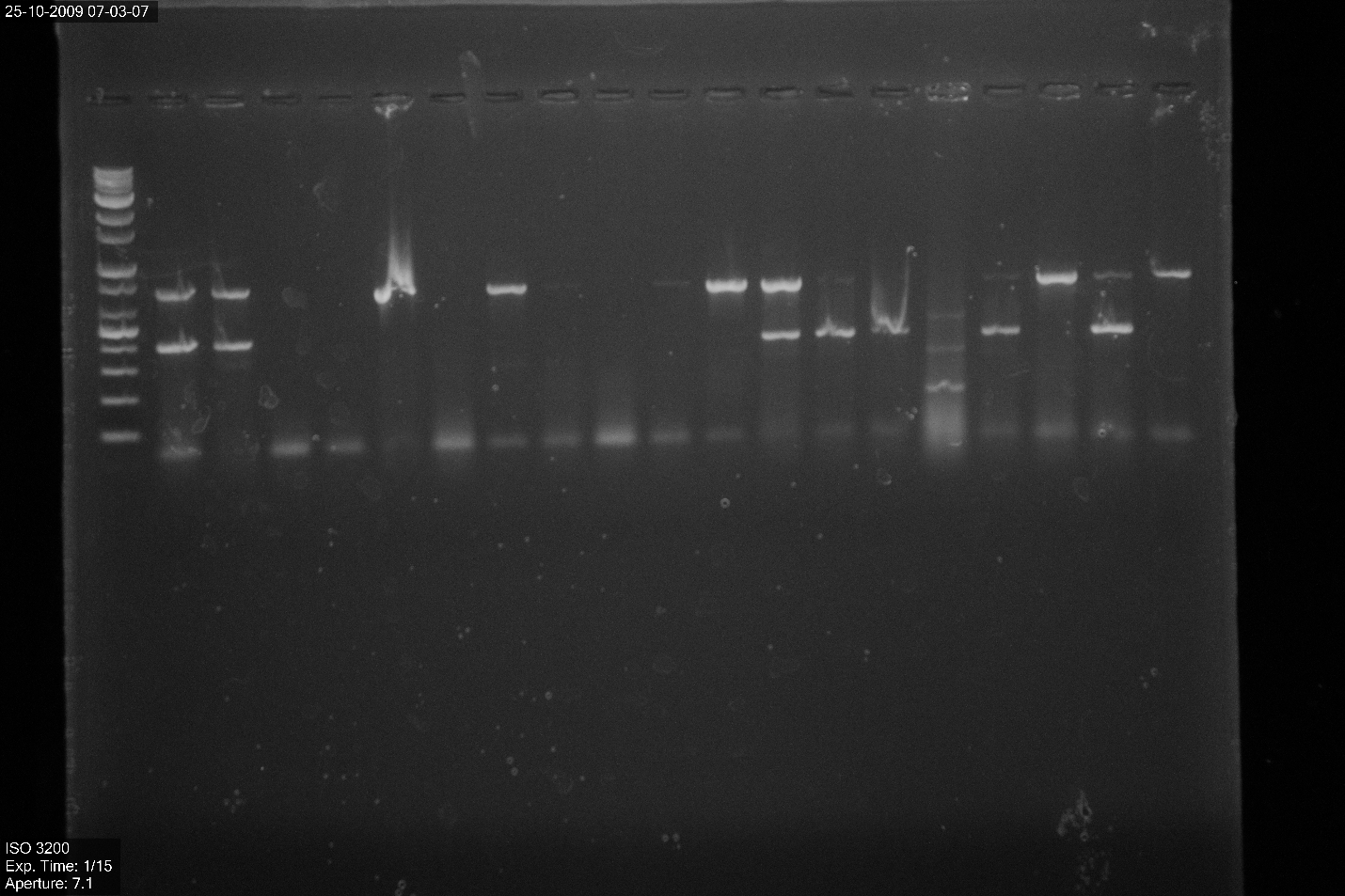


**754 bp**

**404 bp**

M 1 2 3 4 5 6 7 8 9 10 11 12 13 14 15 16 17 18 19

***bla_TEM_***

***bla_CTX-M_***

**Fig 5. Triplex PCR profiles specific for** ***bla_TEM_*, *bla_SHV,_* and *bla_CTX-M_*. Lane M: 10 kb molecular weight marker; lane 1,2,12,18:** ***bla_TEM_* (404 bp) and *bla_CTX-M_* (754 bp); lane 5,7,11,17,19:** ***bla_CTX-M_*** **(754 bp); lane 13,14,16: *bla_TEM_* (404 bp).**


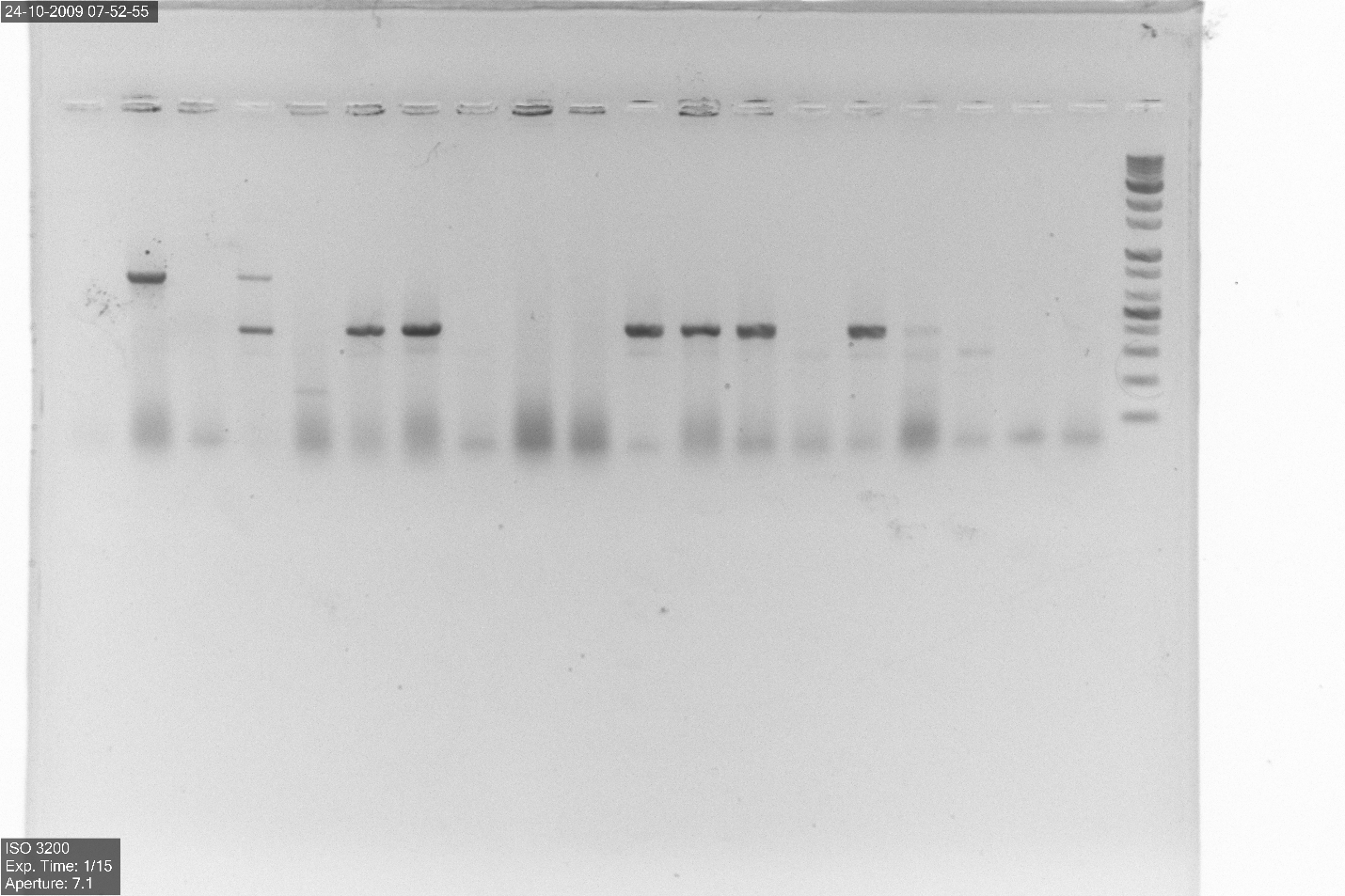


19 18 17 16 15 14 13 12 11 10 9 8 7 6 5 4 3 2 1 M

***bla_OXA-48_***

***bla_NDM_***

**438 bp**

**621 bp**

**Fig. 6. Triplex PCR profiles specific for *bla_KPC_*,** ***bla_NDM,_* and** ***bla_OXA-48_*.** **Lane M: 10 kb molecular weight marker; lane 5,7,8,9,13,14:** ***bla_OXA-48_* (438 bp); lane 16: *bla_OXA-48_* (438 bp) and *bla_NDM_* (621 bp); lane 18*****: bla_NDM_* (621 bp).**


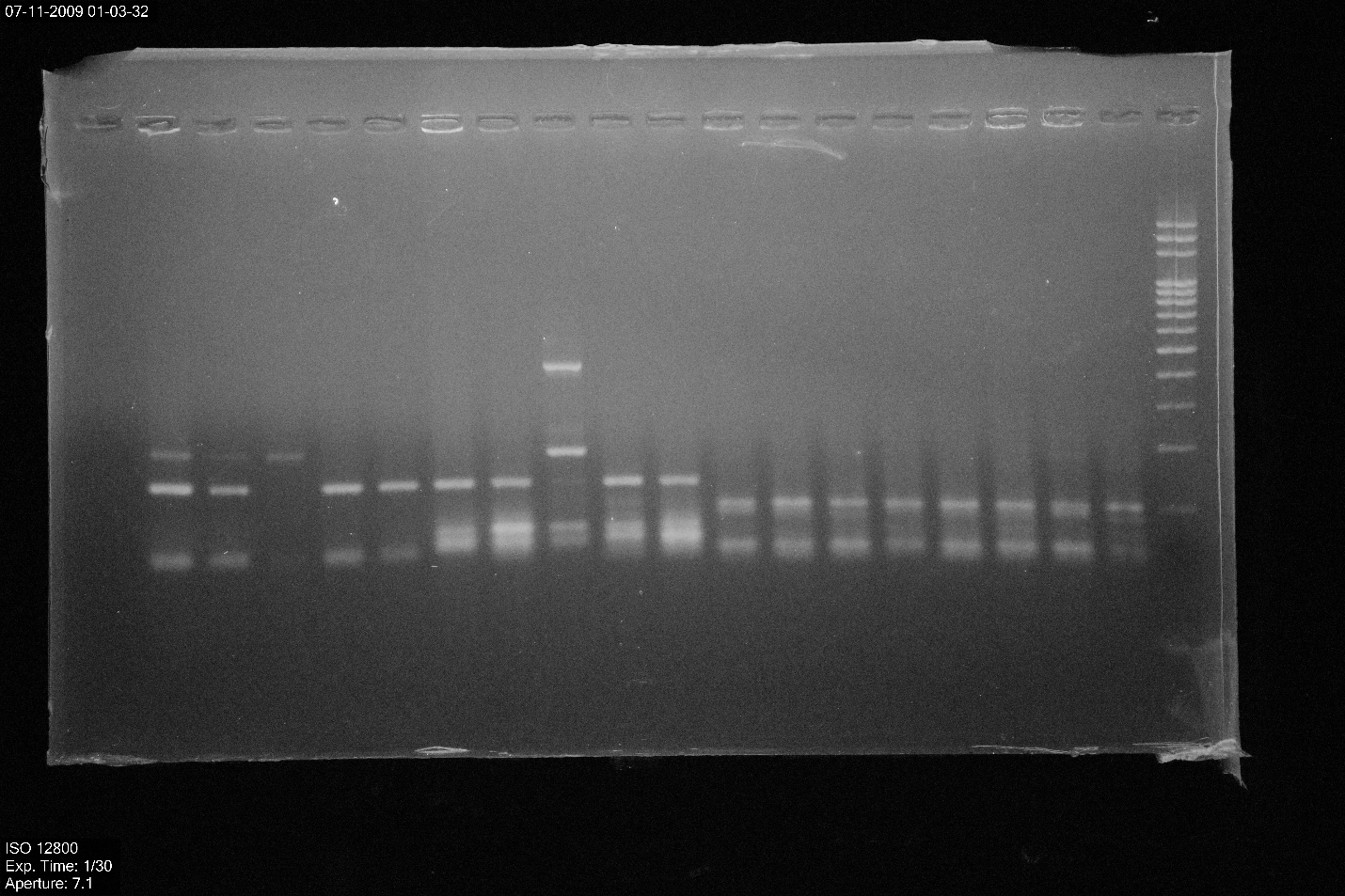


18 17 16 15 14 13 12 11 10 9 8 7 6 5 4 3 2 1 M

**190 bp**

**450 bp**

***LT***

***ST***

**Fig 7. PCR profiles specific for enterotoxigenic *E.coli*.** **Lane M: 10 kb molecular weight marker; lane 11: *LT* (450 bp) and *ST* (190 bp); lane 16, 17, 18: *ST* (190 bp).**
